# Titanosaur eggshells from the latest Cretaceous of Patagonia, Argentina: implications for nesting at high latitude

**DOI:** 10.1098/rsos.260333

**Published:** 2026-09-09

**Authors:** Kohei Tanaka, Fernando E. Novas, Makoto Manabe, Chisako Sakata, Jordi Alexis Garcia Marsà, Darla K. Zelenitsky

**Affiliations:** ^1^ Institute of Life and Environmental Sciences, University of Tsukuba, Tsukuba, Ibaraki Prefecture, Japan; ^2^ CONICET- Fundación de Historia Natural Félix de Azara, Buenos Aires, Argentina; ^3^ National Museum of Nature and Science, Tsukuba, Ibaraki Prefecture, Japan; ^4^ Laboratorio de Anatomía comparada y Evolución de los Vertebrados, Museo Argentino de Ciencias Naturales "Bernardino Rivadavia", Capital Federal, Buenos Aires, Argentina; ^5^ Department of Earth, Energy and Environment, University of Calgary, Calgary, Alberta, Canada

**Keywords:** dinosaur, eggshell, Late Cretaceous, Chorrillo Formation, Argentina, nesting, *Fusioolithus*, titanosaur

## Abstract

Dinosaur egg remains are rarely found in areas of high palaeolatitudes, with Canada (approx. 57–60° N) and Siberia (approx. 70–75° N) yielding the most polarward occurrences. Here, we describe dinosaur eggshells from the Upper Cretaceous (Maastrichtian) Chorrillo Formation of southernmost Patagonia (Argentina), an area located at approximately 55–60° S palaeolatitude during that time interval. The more than 250 eggshell fragments collected are assignable to *Fusioolithus* oosp., an ootaxon known from Late Cretaceous Gondwana regions that probably belongs to titanosaur dinosaurs. The eggshells are shown to have a high porosity like those of titanosaur eggs studied previously, indicating the eggs were fully covered by nest substrate during incubation. The high latitude of the area in the Maastrichtian suggests the eggs were buried in a mound-style nest where microbial decay, rather than solar radiation, was the primary source of incubation heat. The Chorrillo Formation represents, to our knowledge, the highest palaeolatitude area to produce definitive dinosaur egg remains from the Southern Hemisphere and reveals that titanosaurs were nesting at higher latitudes than previously known.

## 1. Introduction

Few discoveries have yielded fossil evidence of non-avian dinosaurs nesting at high palaeolatitudes. Such palaeoenvironments probably posed nesting challenges because of low seasonal temperatures and prolonged periods of reduced sunlight [1,2], where dinosaurs probably would have adopted various reproductive strategies for egg incubation (e.g. early spring nesting, brooding or buried nests) [3,4]. Among non-avian dinosaurs, nesting in northern polar palaeoenvironments is evidenced by hadrosaur and theropod eggshells recovered from a Late Cretaceous site in Siberia representing approximately 70–75° N palaeolatitude [5], as well as theropod perinatal bones and small hadrosaur tracks and perinatal bones from Upper Cretaceous deposits of Alaska [6,7]. Furthermore, fossilized eggshell and perinatal bones of hadrosaur and theropod dinosaurs found in Alberta (Canada) indicate relatively high-latitude nesting at approximately 57–65° N [4,8–13]. While these discoveries indicate nesting at northerly latitudes, less evidence exists for high-latitude nesting in the Southern Hemisphere, in part owing to the lack of definitive dinosaur eggshells/eggs from the regions of Antarctica or Australia.

**Figure 1.**
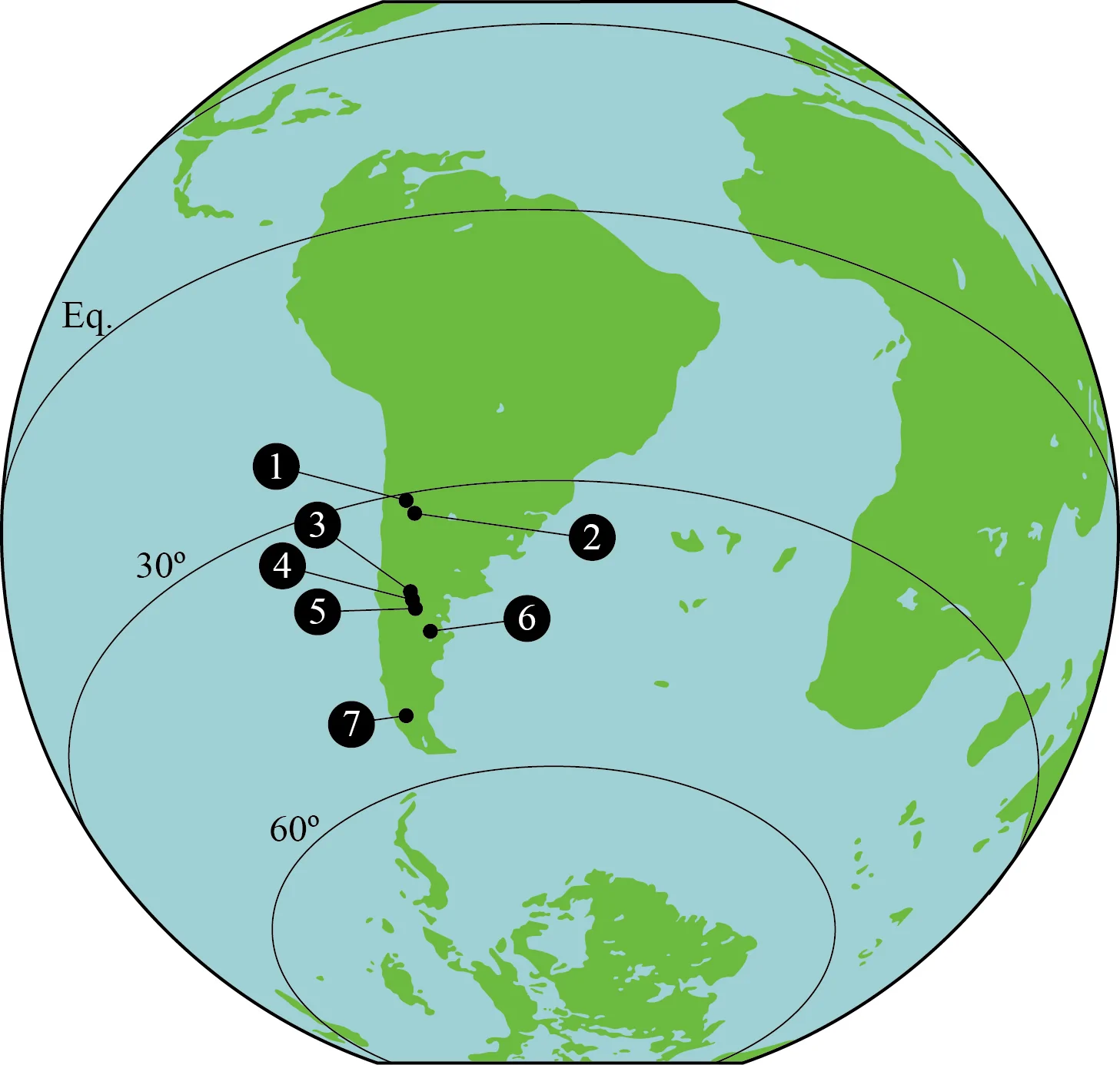
Maastrichtian palaeogeographical map showing Upper Cretaceous localities yielding sauropod eggs/eggshells (oofamilies Faveoloolithidae, Fusioolithidae and Megaloolithidae) in Argentina: (1) Ciénaga Del Río Huaco Formation, (2) Los Llanos Formation, (3) Allen Formation, (4) Bajo De La Carpa Formation, (5) Anacleto Formation, (6) Los Alamitos Formation, and (7) Chorrillo Formation. The palaeogeographical map at 68 Ma was generated using GPlates version 2.5.0 [23] and the global Scotese PALEOMAP [24]. Abbreviation: Eq., equator.

In the Southern Hemisphere, Argentina, a country that has yielded abundant dinosaur eggs, represents a promising region for uncovering fossil evidence of high-latitude nesting. Numerous sites containing eggs/eggshells of mainly sauropod (i.e. titanosaur) dinosaurs (oofamilies Faveoloolithidae, Fusioolithidae and Megaloolithidae) have been found in various Upper Cretaceous rock units, including the Allen [14,15], Anacleto (Río Colorado Subgroup [16,17]), Bajo de la Carpa (Río Colorado Subgroup [18]), Ciénaga del Río Huaco [19], Chorrillo [20], Los Alamitos [21] and Los Llanos formations [22] (figure 1). The palaeolatitudes of the aforementioned sites, except those in the Chorrillo Formation, are reported to represent nesting locations between 26–48° S in the low- to mid-palaeolatitudes [4,19]. Here, we describe, in detail, additional titanosaur eggshell specimens from known sites in the southerly located Chorrillo Formation of Patagonia [20]. Although sauropods have been considered as low-to mid-latitude nesters [4], the Chorrillio Formation specimens extend the range of sauropod nesting to higher palaeolatitudes, providing new insights into the reproductive ecology of titanosaurs inhabiting cool, high-latitude environments.

## 2. Geological and palaeontological context

In recent years, an explosion of palaeontological and sedimentological studies related to the Chorrillo Formation has resulted in a much-improved understanding of this southerly located, Maastrichtian unit. The formation is a roughly 500 m-thick fluvial unit that is underlain by fluvial deposits of the La Irene Formation and overlain by the marine deposits of the Calafate Formation. The age of the Chorrillo Formation has been estimated as late Maastrichtian, based on detrital zircon constraints obtained from the underlying and overlying formations and on its palaeontological record [25]. Channelized and non-channelized deposits comprise the Chorrillo Formation in which fine-grained sediments alternate with coarser-grained conglomeratic/sandstone deposits [26,27]. A variety of non-avian dinosaur fossils (e.g. ankylosaurians, basal euguanodontians, elasmarian ornithischians, probable colossosaurian titanosaurs, hadrosaurids and megaraptorids), birds, crocodylians, turtles, snakes, other reptiles, mammals, frogs, fishes, invertebrates and plant fossils have been recovered from throughout the formation (e.g. [20,26–33]). These sediments and the entombed fossils are considered to have accumulated in a low-gradient fluvial system where high-sinuosity meandering channels and crevasse splays occur within fine-grained (paedogenic) floodplain sediments [26,27]. The region of this Maastrichtian fluvial system has an estimated palaeolatitude of 55–60° S ([34] and calculated here using paleolatitude.org [35]), thus representing a relatively high-latitude ecosystem. Collectively, a diversity of ornithischians, a higher relative abundance of megaraptorid theropods and elasmarian ornithischians and particular fossil monotreme and chironomid arthropod species show parallels with southerly faunas of Antarctica/Australasia [20,26,31,34,36,37], suggestive of transantarctic connections among these regions during the Late Cretaceous [31]. Climatic reconstructions from palynological and palaeosol studies indicate temperate to warm and seasonally humid conditions, with a mean annual temperature of 13.4°C (range 12.1–14.5°C) and an average annual precipitation of 1150 ± 108 mm yr^–1^ (929–1321 ± 108 mm yr^–1^) in this region during the Maastrichtian [25,27,38].

In the Chorrillo Formation, fossilized eggshell is much less common than skeletal remains (e.g. [20]). Previously, eggshell fragments have been recovered from two sites in the lower one-third of the formation. The stratigraphically higher *Magallanodon* site ( = ‘Puma cave’ or ‘Locality 4’) has produced both fusioolithid (*n* > 30) and prismatoolithid (*n* < 10) eggshells, whereas the lower *Kookne* site ( = ‘Titanosaur tibia’ or ‘Locality 2’) has produced only fusioolithid eggshells (see [20] and fig. 1 of [30]). The eggshell fragments studied here were found at the *Magallanodon* site preserved in mudstone/siltstone layers (architectural element consisting of thin dark fine-grained deposits), inferred to represent deposition in a water-logged or swampy environment [26].

## 3. Material and methods

In this study, we examined 255 eggshell fragments (Museo Padre Molina, Río Gallegos, Santa Cruz, Argentina: MPM PV 23560-1 to 23560-255) collected from the Chorrillo Formation during fieldwork in 2020 and 2024. The thickness of the eggshell fragments was measured using a digital micrometre (Mitutoyo CPM30-25 MJ, precision of ± 2 µm). Macro- and microscopic structures were examined via: a binocular microscope (Olympus SZ attached to a digital camera, Nikon D5600); polarized light microscopy (Nikon Eclipse E600POL attached to a digital camera, Canon EOS Kiss X10); scanning electron microscopy (SEM: JEOL JSM-6330F); and micro-computed topography (micro-CT: Shimadzu inspection SMX-225CT FPD HR). Except for the micro-CT scanner at the National Museum of Nature and Sciences in Tsukuba (Japan), all instruments are housed at the University of Tsukuba, Tsukuba. Radial and tangential thin sections were prepared at a thickness of approximately 30 µm. Seven eggshell fragments were coated with platinum before the observations by SEM. Secondary electron images were acquired at an accelerating voltage of 5 kV, with a working distance of approximately 15 mm. For micro-CT analysis, a single eggshell fragment was scanned at a voltage of 225 kV and a current of 70 µA. Digital images acquired via micro-CT scans were processed with Avizo 8.1 (FEI Visualization Sciences Group). Digital images of radial thin sections and SEM were used for the measurements of eggshell structures. All statistical analyses were done with IBM SPSS Statistics v. 29.0.2.0 (IBM SPSS Inc.). Descriptive terminology for the eggshell generally follows [39].

Eggshell porosity was estimated for *Fusioolithus*, following the methodology of Tanaka *et al.* [40]. Briefly, eggshell porosity (mm) is calculated from total pore area in an egg (*A_p_*, mm^2^) divided by pore length (i.e. eggshell thickness, *L*_s_, mm), while assuming the pore canal shape is tubular (see comparative description). Total pore area is a multiplication of single pore area (*A*, mm^2^), density of pores (*D*, mm^−2^) and egg surface area (*A*_egg_, mm^2^). Values of *A* and *D* were obtained from images of tangential thin sections cut at mid-shell thickness (*n* = 5) using Adobe Photoshop 25.12.0 and ImageJ 1.53e (W. Rasband, National Institutes of Health, Bethesda, Maryland, USA). *A*_egg_ is unknown for *Fusioolithus* eggshells from the Chorrillo Formation owing to a lack of complete eggs. Thus, we used the minimum and maximum egg sizes of *Fusioolithus* from previous studies (supplementary material, table S1); the diameter of the minimum (L, 110 mm) and maximum (230 mm) egg sizes were taken from [41] and [42], respectively. Assuming the egg was spherical, we estimated the minimum and maximum *A*_egg_ (see table 3 of [40]). In addition, the minimum and maximum egg mass (*M*, g) were calculated using an equation of Hoyt [43]: 5.48∙10^−4^∙L^3^ (for spherical eggs), which was derived from extant bird eggs. Then, an ordinary least-squares regression of eggshell porosity against estimated egg mass was conducted. A linear discriminant analysis based on the eggshell porosity and egg mass dataset provided by Tanaka *et al.* [40] was conducted to infer the nest type (covered nest type or open nest type, the latter meaning at least partially open) for a particular ootaxon. The statistical analysis was described in detail by Tanaka *et al.* [40].

Nesting styles and incubation heat sources of titanosaur (sauropod) oofamilies (Faveoloolithidae, Fusioolithidae and Megaloolithidae) were inferred and compared using their preferences of nest substrates (i.e. entombing sediments of *in situ* fossil nests); the methodology is explained in detail by Tanaka *et al.* [4]. In brief, one-way *χ*^2^-tests were used to test if each oofamily was associated with a certain type of (nest) sediment. Nest sediments for each entry were categorized as fine-grained (i.e. siltstone and mudstone, including marls) or coarse-grained (i.e. sandstone and conglomerate), but limestone was not included in the dataset. Palaeosol/pedogenic features (e.g. bioturbation, caliche nodules, pedotubules, rhizoliths, root traces and slickensides) were also recorded for each entry. Thus, here we updated the dataset of Tanaka *et al.* [4].

For Faveoloolithidae, Fusioolithidae and Megaloolithidae ootaxa, we also recorded their global palaeogeographical distribution from the literature and estimated their palaeolatitudes using the website paleolatitude.org [35], updating the dataset of Tanaka *et al.* [4]. Box plots of absolute palaeolatitudes were produced for each oofamily.

## 4. Systematic palaeontology

Oofamily Fusioolithidae [15]

Oogenus *Fusioolithus* [15]

Oospecies *Fusioolithus* oosp. indet.

Referred specimens—isolated eggshell fragments (*n* = 255) (MPM PV 23560-1 to 23560-255).

Locality, horizon and age: eggshell specimens examined in this study were recovered from mudstone/siltstone layers at the *Magallanodon* site (50°30′38.59′′ S, 72°33′37.84′′ W), a site located approximately 29 km southeast from El Calafate, Santa Cruz Province, Patagonia, Argentina. The *Magallanodon* site is the lower third of the Upper Cretaceous (probably lower Maastrichtian) Chorrillo Formation.

**Figure 2.**
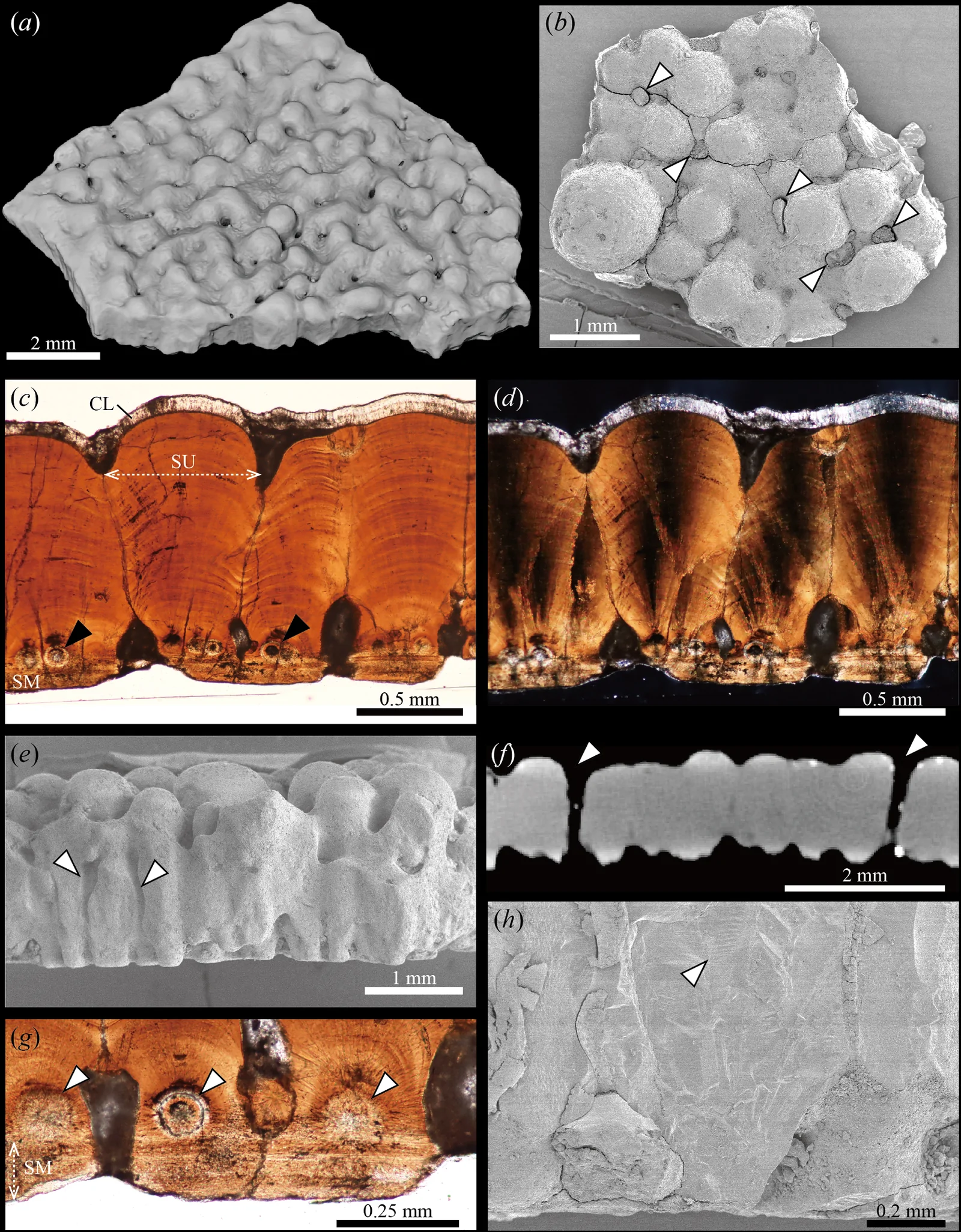
*Fusioolithus* oosp. from the Chorrillo Formation, southernmost Patagonia: (*a*) rendered image of eggshell micro-CT data (MPM PV 23560-3), showing compactituberculate ornamentation; (*b*) eggshell outer surface under SEM (MPM PV 23560-2), showing pore openings (arrows); (*c*) radial thin section of eggshell under plane polarized light (MPM PV 23560-94), showing spatula-shaped shell unit (SU) and organic core (black arrows) with secondary calcite layer (CL) and shell membrane (SM); (*d*) radial thin section of (*c*) under cross-polarized light, showing sweeping extinction pattern; (*e*) radial view of eggshell under SEM (MPM PV 23560-254), showing transversely-merged shell units and pore canals (arrows); (*f*) micro-CT slice of eggshell in radial view (MPM PV 23560-3), showing relatively straight pore canals (arrows); and (*g*) radial thin section shell unit base under plane polarized light (MPM PV 23560-94), showing organic core (white arrows) and finely-laminated shell membrane (SM); and (*h*) base of shell unit under SEM (MPM PV 23560-2), showing blocky, tabular crystals (arrows).

**Figure 3.**
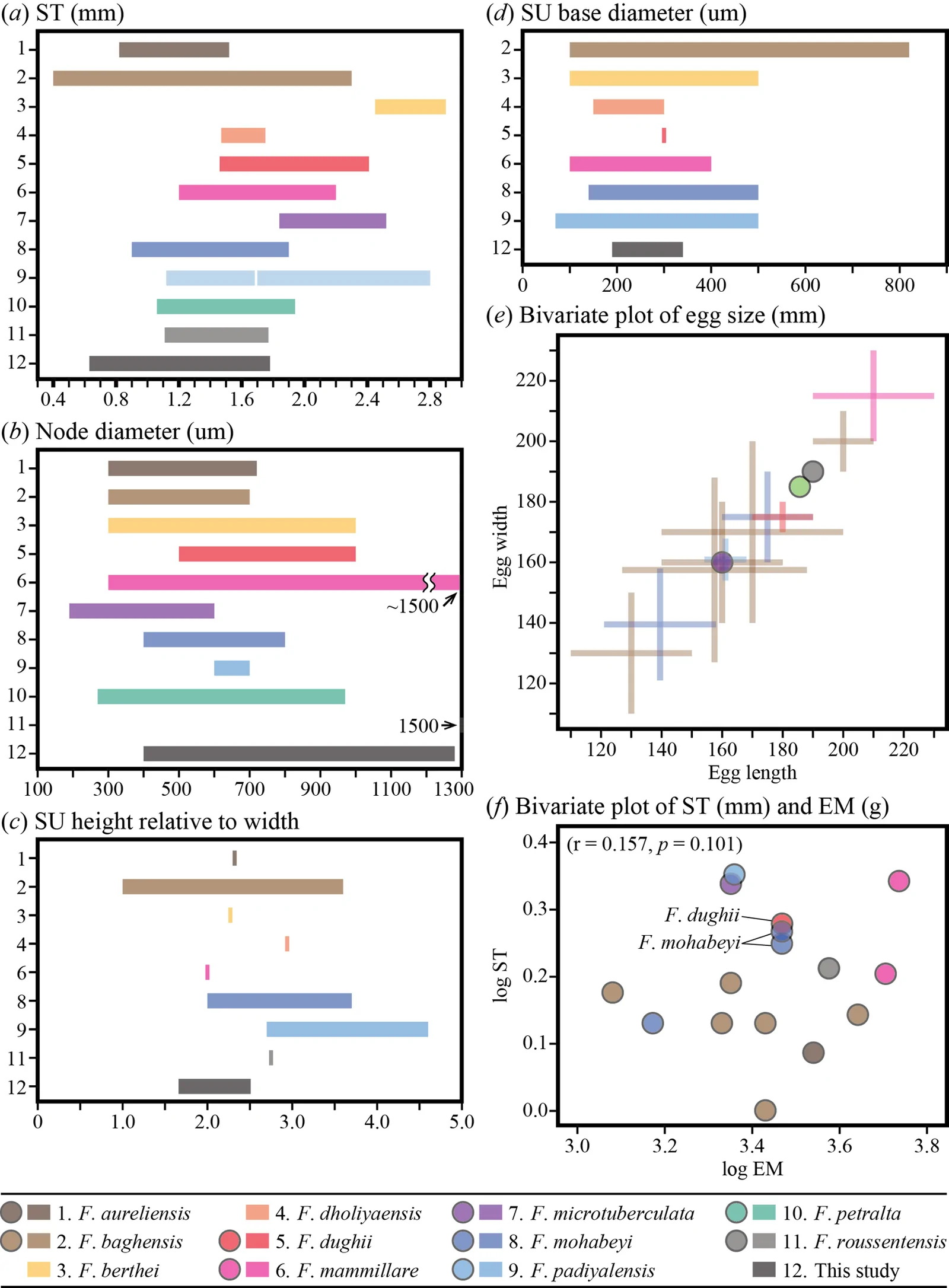
Comparisons among *Fusioolithus* oospecies: (*a*) eggshell thickness; (*b*) single node diameter; (*c*) height/width ratio of eggshell unit; (*d*) eggshell unit base diameter; (*e*) bivariate plot of egg length and width; and (*f*) bivariate plot of eggshell thickness and egg mass in 10-based logarithmic scale, showing no significant correlation. Abbreviations: EM, egg mass; ST, eggshell thickness and SU, shell units.

Comparative description: the eggshell fragments (*n* = 255) are relatively small in size, up to 133 mm^2^ in surface area (figure 2*a*,*b*). The eggshell thickness, measured using callipers (255 fragments measured), ranges from 0.63 to 1.78 mm (mean value, 1.17 mm), excluding the height of the ornamentation (figure 3*a*). These values indicate that, of the two valid Fusioolithidae oogenera, the eggshell is assignable to *Fusioolithus* rather than to the overall thinner *Litosoolithus* (0.8–1.1 mm [44]).

The outer surface displays a compactituberculate ornamentation, typical of *Fusioolithus*, where the nodes appear circular shaped in plan view (node diameter, 0.40–1.28 mm; mean value, 0.66 mm: figure 3*b*), although the nodes sometimes coalesce (figure 2*a*,*b*); *Litosoolithus* differs in having dispersituberculate ornamentation with irregularly distributed bell-shaped nodes [44]. In some eggshells, the outer surface is covered with a thin, secondary calcitic layer owing to diagenesis (figure 2*c*,*d*). Circular to semicircular pore openings occur in the lows between the nodes and are approximately 0.15–0.40 mm in diameter (figure 2*b*).

The eggshell structure, consisting of a single layer of fan- or spatula-shaped shell units, is assignable to the tubospherulitic morphotype (figure 2*c*–*e*). The shell units are taller than wide, with a height-to-width ratio ranging from 1.66 : 1 to 2.51 : 1 (mean = 2.16 : 1), which overlaps with all other *Fusioolithus* oospecies, except for both *Fusioolithus dholiyaensis* and *Fusioolithus padiyalensis* that have greater height-to-width ratios (figure 3*c*). In radial thin section, fine accretion lines are laterally continuous across the shell units and undulate in correspondence with the nodes (figure 2*c*), which is not found in *Litosoolithus*. Under cross-polarized light, the boundaries between shell units are more evident owing to the sweeping extinction pattern (figure 2*d*). Shell units are separated in the lower one-half to one-third of the eggshell, whereas they are coalesced, at least in part, in the upper portion. Numerous tubocanaliculate-type pore canals (mean pore density, 3.17 mm^–2^) exist between the shell units and are mostly tubular and unbranching, with some slightly curved or angled (figure 2*e*,*f*). Only two of 47 canals observed using micro-CT were branched, but the branches did not pierce the outer surface. The pore canals are circular to oval in cross-sectional shape, and their diameters increase near the outer and inner surfaces of the shell.

The shell unit bases are visibly composed of blocky, tabular crystals in radial view and are circular to rectangular in plan view (diameter, 0.19–0.34 mm; mean, 0.28 mm: figures 2*h* and 3*d*). Occasionally, multiple small basal caps are fused together within an individual shell unit (figure 2*c*), similar to those of *F*. *dholiyaensis* and *F*. *padiyalensis* [15,45]. Organic cores, located at the base of the shell units, and attached partial shell membranes are visible in some well-preserved eggshell fragments, suggesting rapid burial of eggs ([46]: figure 2*c*,*d*,*g*). The organic cores are spherical (0.08–0.17 mm in diameter), and the attached remnants of shell membranes are finely laminated and about 0.11 mm in thickness (figure 2*g*).

Remarks: the Chorrillo eggshells examined in this study are assignable to the oofamily Fusioolithidae based on the presence of the tubospherulitic morphotype, compactituberculate ornamentation and tubocanaliculate pore system, as well as the presence of partially-fused, fan-shaped shell units with accretion lines that laterally extend across shell units (supplementary material, table S1). Fernández & Khosla [15] proposed to establish the oofamily Fusioolithidae, along with two new oospecies of *Fusioolithus* (i.e. *Fusioolithus baghensis* and *Fusioolithus berthei*), and suggested the reassignment of several oospecies previously assigned to Cairanoolithidae and Megaloolithidae to *Fusioolithus* (i.e. *Cairanoolithus dughii*, *Cairanoolithus roussetensis*, *Megaloolithus aureliensis*, *Megaloolithus dholiyaensis*, *Megaloolithus mammillare*, *Megaloolithus microtuberculata*, *Megaloolithus mohabeyi*, *Megaloolithus padiyalensis* and *Megaloolithus petralta*) because they also have fused adjacent shell units. However, some of these ootaxonomic assignments remain controversial and may require further revision (e.g. [47,48]). For example, *Fusioolithus petralta* could be a junior synonym of *Fusioolithus aureliensis* [47], whereas *Cairanoolithus* may be distinguishable from *Fusioolithus* based on its columnar shell units and poorly ornamented eggshell surface [48].

The Chorrillo eggshells and other *Fusioolithus* oospecies are very similar to one another in terms of eggshell thickness (except *F*. *berthei* and *Fusioolithus microtuberculata*), node diameter, shell unit height to width ratio (except *F.dholiyaensis* and *F.padiyalensis*), shell unit base diameter and egg size (when preserved) (figure 3). Thus, despite ongoing debate regarding the status of some referred ootaxa, the close similarities among recognized *Fusioolithus* oospecies and the difficulty of differentiating their diagnoses preclude a confident assignment of the Chorrillo eggshells to a particular oospecies in the formation.

Skeletal remains of titanosaurs are known from the Chorrillo Formation (Titanosauria indet. and ?colossosaurian *Nullotitan glaciaris* [20,26]); titanosaur teeth have been recovered from the same site (i.e. *Magallanodon* site) as the eggshells studied here. Although there is no direct association between skeletal remains and eggshells, the eggshells could have been laid by titanosaur taxa represented by skeletal remains.

## 5. Analyses of nest type and substrate

**Figure 4.**
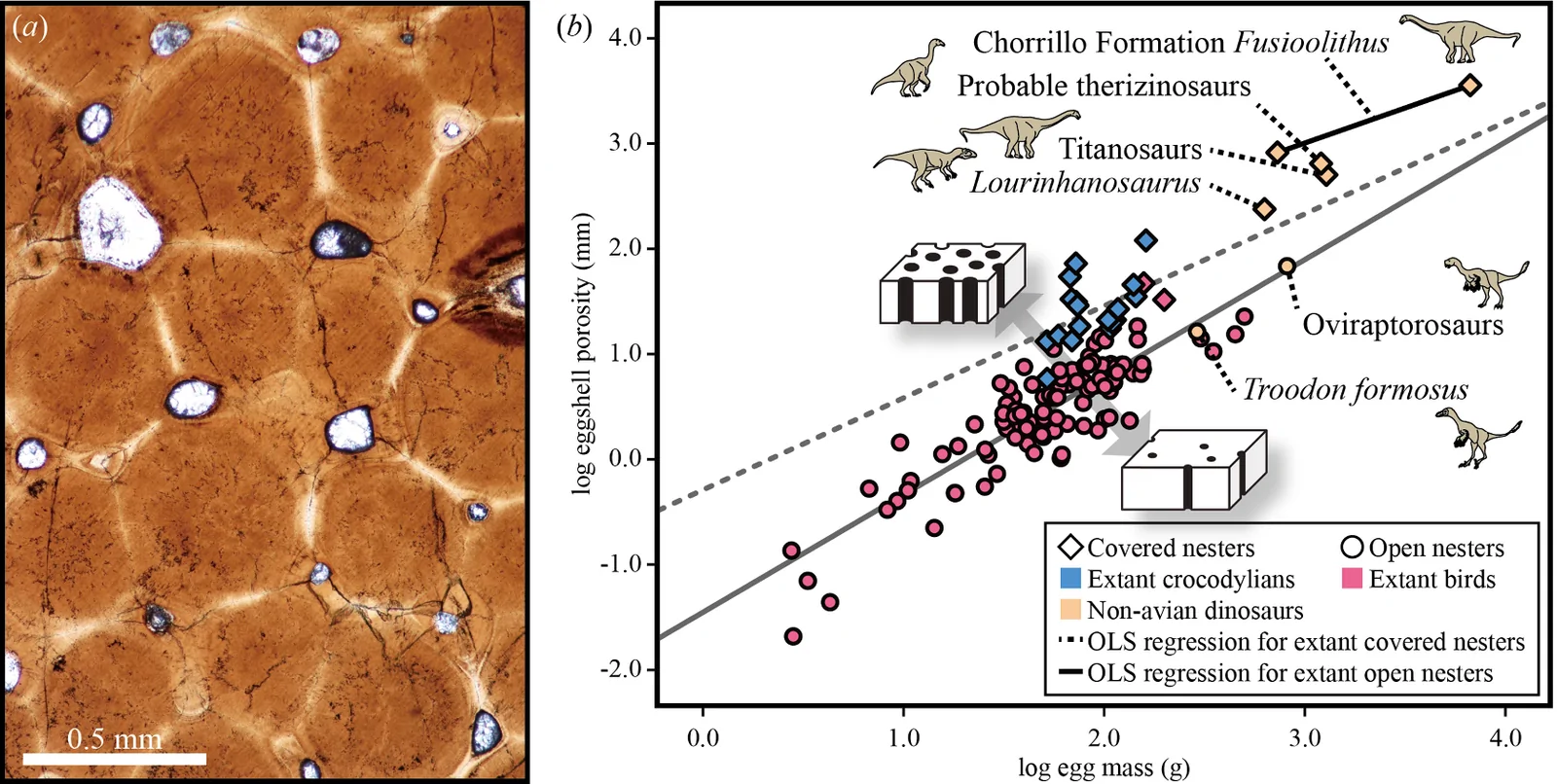
Porosity analysis of *Fusioolithus* oosp: (*a*) tangential thin section of eggshell under plane polarized light (MPM PV 23560-179), showing porous eggshell; and (*b*) bivariate plot of eggshell porosity and egg mass in 10-based logarithmic scale, showing porosity of *Fusioolithus* (orange line) is comparable to that of extant covered nesters and titanosaurs, *Lourinhanosaurus* and probable therizinosaurs. Abbreviation: OLS, ordinary least squares.

Eggshell porosity estimations: minimum (110 mm) and maximum (230 mm) egg diameter values for spherical *Fusioolithus* eggs (supplementary material, table S1;  figure 3*e*,*f*) yielded values of 729.34 and 6667.52 g for egg mass, respectively. With a pore density of 3.17 mm^–2^ and a single pore area of 8006.18 µm^2^, eggshell porosity (= total pore area divided by pore length) of these eggs is estimated at 821.15 mm and 3584.70 mm, respectively. Because the single pore area was calculated at the midpoint of the eggshell thickness, where the pore diameter is probably smallest, the resulting porosity values represent minimum estimates. Nevertheless, regardless of egg size, linear discriminant analyses reveal that the Chorrillo *Fusioolithus* eggs are highly porous, grouping with eggs that are incubated in a covered nest type with >0.998% posterior probabilities (figure 4).

The estimated eggshell porosity values of the Chorrillo *Fusioolithus* eggs fall within or above the range reported for other fusioolithid and megaloolithid eggs (including cairanoolithids), as well as other titanosaur eggs (287.16–2125.21 mm) considered to have been incubated in covered nests [40].

**Figure 5.**
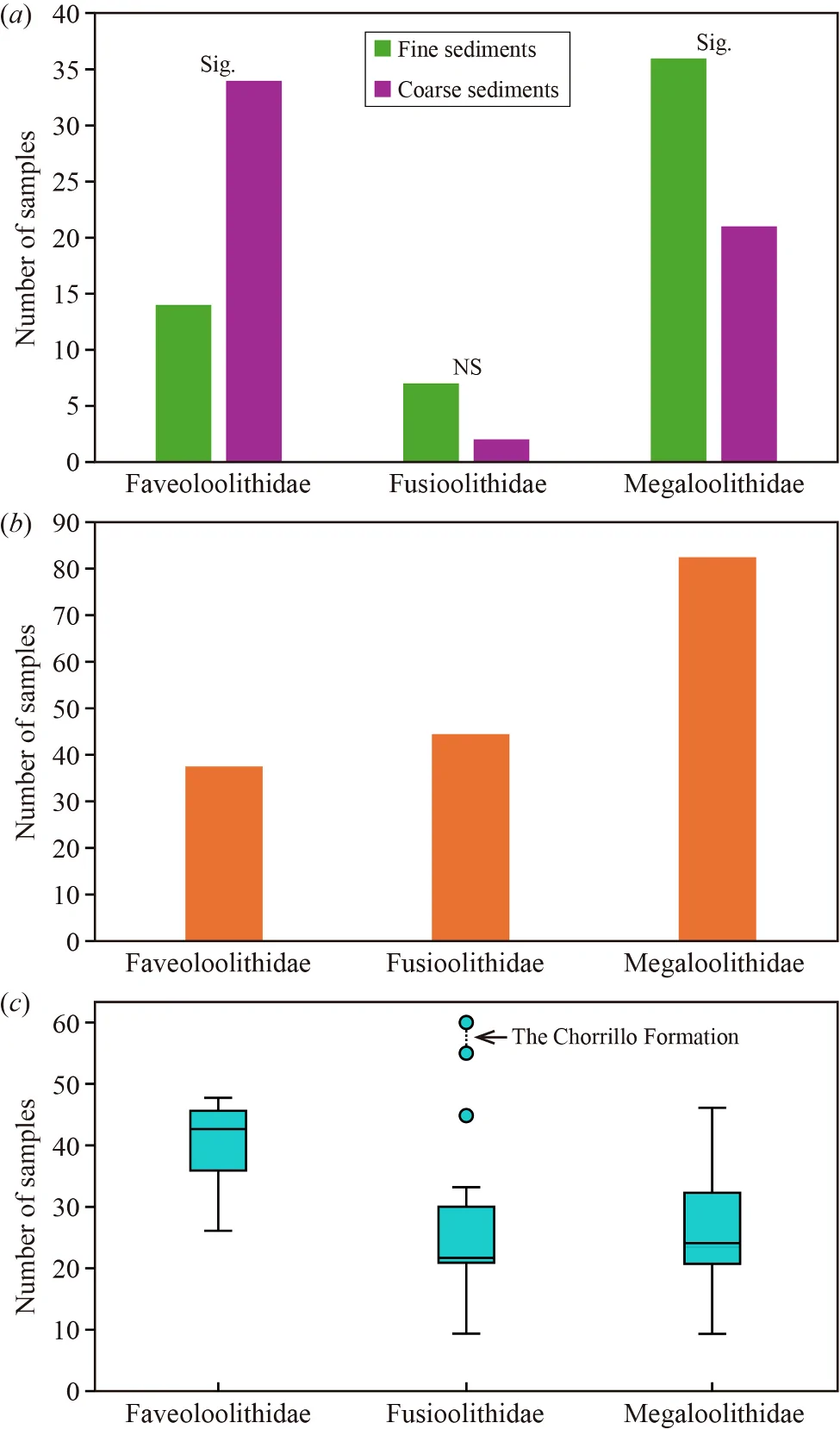
Comparison of sediment types among sauropod oofamilies: (*a*) comparison of *in situ* clutch lithology, showing clutches of Faveoloolithidae and Megaloolithidae tend to be associated with particular sediments whereas those of Fusioolithidae detected no preferential association with lithology; (*b*) relative abundance of palaeosol or pedogenic features found in clutches, showing that clutches of Megaloolithidae are strongly associated with palaeosols, whereas the other two oofamilies are not; and (*c*) comparison of absolute palaeolatitudes in sauropod egg/eggshell localities, showing fusioolithid nesting sites include relatively high latitude compared with the other two oofamilies. Abbreviations: sig., significance and NS, no significance.

Nest substrate associations: the one-way *χ*^2^-tests of nest sediments for Faveoloolithidae, Fusioolithidae and Megaloolithidae demonstrate that Faveoloolithidae and Megaloolithidae preferred specific nest substrates (table 1;  figure 5*a*). Nests of Faveoloolithidae are associated with coarse sediments rather than fine ($\chi_{1}^{2}$ = 8.333, *p* <0.05), whereas those of Megaloolithidae are associated with fine sediments rather than coarse ($\chi_{1}^{2}$ = 3.947, *p* <0.05); these results are concordant with those of Tanaka *et al.* [4]. Probably owing to the small sample size (*n* = 9), no significant result was obtained for Fusioolithidae (*p* = 0.096), although their nests are found in fine sediments more often than in coarse sediments.

**Table 1.** Results of one-way *χ*^2^-tests for the *in situ* datasets of sauropod oofamilies. (Abbreviations: d.f., degree of freedom; *n*, sample size; NA, not applicable; *p*, probability for *χ*^2^-tests.)

oofamily	*n* for fine-grained	*n* for coarse grained	total *n*	expected *n* for fine-grained	expected *n* for coarse-grained	*χ*^2^ value	d.f.	*p*
Faveoloolithidae	14	34	48	24.0	24.0	8.333	1	0.004
Fusioolithidae	7	2	9	4.50	4.50	2.778	1	0.096
Megaloolithidae	36	21	57	28.5	28.5	3.947	1	0.047
total		63	118	NA	NA	NA	NA	NA

Evidence of palaeosols and/or pedogenic features is common in Megaloolithidae (82.46%) but relatively rare in Fusioolithidae (44.44%) and Faveoloolithidae (37.50%) (figure 3*b*); however, the sample size for Fusioolithidae is small (*n* = 9).

## 6. Discussion

Although titanosaur skeletal remains are known from high-southern palaeolatitudes (e.g. southern Patagonia, Antarctica and Australia [20,49,50]), prior egg discoveries indicate that these dinosaurs nested primarily in low- to mid-palaeolatitude regions (average approx. 31°: table 2; figure 3*c* ; supplementary material, table S3). Here, we describe, in more detail, probable titanosaur eggshells of the oogenus *Fusioolithus* (oofamily Fusioolithidae) from sites in the Maastrichtian Chorrillo Formation of southernmost Argentina, an area located at approximately 55–60° S palaeolatitude ([34] and calculated here using paleolatitude.org [35]). Including the eggshells described by Novas *et al.* [20], these fossil occurrences provide, to our knowledge, the first direct evidence that titanosaurs—a clade of the largest known dinosaurs—were nesting at relatively high latitudes in the latest Cretaceous.

**Table 2.** Ranges of absolute palaeolatitudes for sauropod oofamilies. (Abbreviation: *n*, sample size.)

oofamily	*n*	range of absolute palaeolatitudes
Faveoloolithidae	32	26.10° to 47.73°
Fusioolithidae	30	9.35° to 55–60°
Megaloolithidae	48	9.35° to 46.12°

Global occurrences of titanosaur eggs (oofamilies Faveoloolithidae, Fusioolithidae and Megaloolithidae) reveal some patterns in their geographical distribution. Faveoloolithidae is known from East Asia and South America, whereas Fusioolithidae and Megaloolithidae are known from Europe and former Gondwanian regions of Africa (only Megaloolithidae, one occurrence), India and South America. On average, Faveoloolithidae eggs occur at higher (absolute) palaeolatitudes (41°) than Fusioolithidae (28°) and Megaloolithidae (26°) eggs (figure 5*c*). Although the highest occurrence of Faveoloolithidae egg remains are from Argentina and Mongolia at approximately 48° N palaeolatitude, the Chorrillo Fusioolithidae eggshells, at 55–60° S, are the highest for all titanosaur eggs.

*Fusioolithus* eggshells from the Chorrillo Formation possess a high eggshell porosity, indicating that these eggs were incubated in covered nests buried by nesting materials, as in Faveoloolithidae and Megaloolithidae [4]. Such covered nests are built by some living archosaurs (crocodilian and megapode bird species) in which the eggs are incubated using heat from microbial decay (fermentation of plant/organic matter), solar radiation or geothermal activity. Although the incubation heat source can be predicted for such nests from correlations of nest sediments/substrates in living archosaur species [4], the limited sample size for fusioolithid eggs precludes a reliable prediction. Thus, the specific incubation heat source used for fusioolithid eggs from the Chorrillo Formation remains uncertain, although microbial decay of nest vegetation remains a plausible source as solar incubation is generally limited to lower latitudes [4]; there is currently no evidence of geothermal activity preserved in the area, unlike that reported for the Los Llanos Formation [51]. Adequate incubation heat can be produced by nest (vegetation) fermentation even in cooler environments where ambient temperatures are only approximately 15°C [4]; the Chorrillo Formation (55–60° S) region had an estimated mean annual palaeotemperature of 12–14.5°C [25], probably able to yield adequate ambient temperatures seasonally for such nesting styles.

As indicated by the occurrence of thin prismatic eggshells previously described from the Chorrillo Formation (referred to as Prismatoolithidae in [20]), an alternate incubation heat source was potentially used by small non-avian theropods/birds nesting in the region. Although the eggshell porosity of these few prismatic eggshells has not been estimated to predict nest type, no pores are evident from the eggshell description [20], indicating a low eggshell porosity and thus egg incubation in an open nest type, like Paraves and nearly all bird species [40]. If the eggshell porosity is truly low, then such eggs would have been at least partially exposed (open nest) and a brooding adult probably provided incubation heat.

The Chorrillo Formation represents a rich and diverse flora and fauna in climatic conditions that were temperate to warm and seasonally humid [20,25–27,34,38]. Dinosaurs, birds, mammals and other vertebrates inhabited the region, some of which nested there, as evidenced by fossilized eggshell occurrences. The presence of titanosaur (*Fusioolithus*) and small non-avian theropods/birds (prismatic-type) eggshells [20] indicates that these dinosaurs were nesting in this high-latitude area, probably via covered nests and brooding adults, respectively.

The Chorrillo Formation has yielded the highest latitude occurrence of definitive dinosaur egg remains in the Southern Hemisphere. Although it is difficult to compare eggshell localities of the Chorrillo Formation to other such high palaeolatitude sites since so few exist, probably the most comparable would be those of the Horseshoe Canyon Formation area of southern Alberta, Canada. Both the Chorrillo and Horseshoe Canyon formations, although in very different regions with differing dinosaur-dominated faunas and floras, represent temperate/warm and seasonally humid [10,27], Maastrichtian ecosystems that preserve eggshell at similarly high (absolute) palaeolatitudes. The area of the Horseshoe Canyon Formation, at approximately 60° N palaeolatitude [10], has also yielded prismatic eggshell fragments belonging to small theropods [11], as well as perinatal bones of theropods and hadrosaurs [52]. Like the area of the Chorrillo Formation, covered nests (using microbial decay) and adults brooding open nests have been inferred/predicted as incubation heat sources for such dinosaurs nesting in Alberta [4]. We anticipate that future discoveries of egg fossils at higher palaeolatitudes in Argentina, Canada and elsewhere will undoubtedly further our understanding of dinosaur egg incubation and nesting at cooler/higher latitudes.

## 7. Conclusion

Fossil evidence of dinosaurs nesting at high palaeolatitudes is scarce in the Southern Hemisphere. Probable titanosaur eggshells, assignable to the ootaxon *Fusioolithus* oosp., recovered from the Maastrichtian Chorrillo Formation of southernmost Patagonia, represent the highest palaeolatitude (55–60° S) evidence known from the Southern Hemisphere. These eggshells provide, to our knowledge, the first indication that titanosaurs nested at relatively high latitudes. The eggs, as shown by a high eggshell porosity, would have been incubated in nests buried by nesting materials and would have used the heat generated by decaying vegetation. A similar nesting style has also been inferred for non-brooding dinosaurs in high-northern palaeolatitudes, where sufficient heat from solar radiation was probably  unavailable.

## Supplementary Material



## Data Availability

The data are provided in the electronic supplementary material [53].
