# Supplementary material for: Titanosaur eggshells from the latest Cretaceous of Patagonia, Argentina: implications for nesting at high latitude
Source: R Soc Open Sci. 2026 Sep 9;13(9):260333. doi: 10.1098/rsos.260333 (PMC13561085; doi:10.1098/rsos.260333)
Supplement: Supplementary file 1 [file rsos.260333.zip › rsos.260333/rsos260333_si_001.docx]

Titanosaur eggshells from the latest Cretaceous of Patagonia, Argentina: implications for titanosaur nesting at high latitude

Kohei Tanaka, Novas Fernando, Makoto Manabe, Chisako Sakata, Jordi Garcia, Darka K. Zelenitsky

Supplementary Information

Contents

**Table S1**. Comparisons of fusioolithid ootaxa.

**Table S2**. Lithology of in-situ sauropod eggs, clutches and nesting grounds (Faveoloolithidae, Fusioolithidae, and Megaloolithidae).

**Table S3**. Approximate paleolatitudes of sauropod egg localities.

**References**

**Table S1**. Comparisons of fusioolithid ootaxa. Asterisks indicate ootaxa that were previously assigned to *Megaloolithus*. Values within parentheses indicate means or main ranges. ⁑Eggshell thickness was calculated here based on Figure 1 of Lourembam et al. (2022). † indicates the eggshell thickness including the height of ornamentation whereas ‡ indicates the eggshell thickness excluding the height of ornamentation; the other measurements of eggshell thickness were unknown whether the height of ornamentation is included or excluded. Abbreviation: SU, shell unit.

| Ootaxon | Formation | Locality | Egg diameters (mm) | Eggshell thickness (mm) | Node diameter (um) | Ratio of SU height to width | SU base diameter (um) | References |
| --- | --- | --- | --- | --- | --- | --- | --- | --- |
| *Fusioolithus* *aureliensis** | Aix-en-Provence (upper Campanian-lower Maastrichtian); Tremp Formation (Maastrichtian) | Clos-La-Neuve, Roquehautes-Grand Creux-A, B, and D, Villeveyrac Basin, and Saint-Chinian, Aix-en-Provence, France; Coll de Nargo, Lleida, Catalonia, Spain | 185; 194 x 100? (deformed) | 0.82–1.52; 0.90–1.10; 1.12–1.32; 1.11–1.44 (1.29) | 300–700 (500); 500; 300–720; 300–700 | 2.32:1 | ? | Vianey-Liaud et al. (1994); Sellés et al. (2013) |
| *F*. *baghensis** | Lameta Formation (Maastrichtian); Intertrappean beds (Maastrichtian); Fundo El Triunfo Formation (late Campanian to Maastrichtian?); Allen Formation (Campanina-Maastrichtian); Red clay beds of the Aix-en-Provence Basin (latest Maastrichtian); Villalba de la Sierra Formation (Campanian-Maastrichtian) | Rahioli, Balasinor Taluka, Jetholi, and Dhuvadiya of Kheda, Gujarat; Bagh Cave Section, Pisdura, Akhada, Jhaba, Jamniapura, and Padlya of Dhar and Lametaghat in Jabalpur, Madhya Pradesh; Piplanarayanwar, Chhindwara, Madhya Pradesh; Pisdura, Chandrapur, Maharashtra, India; Laguna Umayo, Bagua Basin, Peru; Salitral Moreno, Rio Negro Province, Argentina; Les Breguieres, Aix-en-Provence, France; Poyos fossil site, Guadalajara Province and Zafra de Zancara, Cuenca Province, Castilla-La Mancha, Spain | 110–150; 140–200; 140–180; 127–188; 190–210 | 1.50; ~1.00; 1.00–1.70; 1.45–1.65; 0.40–2.30; 0.72–0.85⁑, †; 0.60–0.72⁑, ‡; 0.60–1.17 (1.00–1.17); 1.05–1.61 (1.56); 1.03–1.99 (1.21–1.57); 1.00–1.40 (1.10) | 300–700 (410) | 1.00:1; 1.20–3.60:1 (2.34:1); 1.60:1; 2.28:1; 2.32:1; 2.50:1 | 150–300; 200–300; 100–400 (282); 180–370; 150–820 (310) | Mohabey (1984, 1998); Srivastava et al. (1986); Vianey-Liaud et al. (1994, 1997, 2003); Khosla and Sahni (1995); Simon (2006); Dhiman et al. (2022, 2023); Lourembam et al. (2022); Sanguino et al. (2025) |
| *F. berthei* | Allen Formation (Campanina-Maastrichtian) | Salitral de Santa Rosa and Salitral Ojo de Agua, Rio Negro Province, Argentina | ? | 2.60–2.80; 2.45–2.90 | 300–1000; 300–700 (660) | 3.27:1 | 400–500; 100–500 | Fernández (2013); Fernández and Khosla (2015) |
| *F*. *dholiyaensis** | Lameta Formation (Maastrichtian) | Dholiya, Dhar, Madhya Pradesh, India | ? | 1.47–1.75 | ? | 2.94:1 | 150–300 | Khosla and Sahni (1995) |
| *F.* *dughii* | Aix-en-Provence (Maastrichtian); Red Marls of Maurine Formation (Maastrichtian) | La Cairanne, Roquehautes-Grand Creux, Villeveyrac Basin, and Commune de Rousset, Aix-en-Provence, France; Les Boudous and Gourg de l'Encantado, Upper Aude Valley, France | 170-180 | 1.57-2.41; 1.90; 1.56-2.02 (1.86); 2.02-2.23 (2.13) | 500-1000 | ? | 300 | Williams et al. (1984); Vianey-Liaud et al. (1994); Fondevilla et al. (2016) |
| *F.* *mammillare** | Aix-en-Provence (Maastrichtian); Red Marls of Maurine Formation (Maastrichtian) | Rousset-Erben, La Cardeline, Les Breguieres, Commune de Rousset, and Commune de Chateauneuf Le Rouge, Aix-en-Provence, France; Roque Fumade, Upper Aude Valley, France | 190–230; 200–230 | 1.20–2.10; 2.20; 1.60; 1.52–2.17 (1.89) | 300–1200 (1000); ~1500 | ~2.00:1 | 100–400 | Williams et al. (1984): Vianey-Liaud et al. (1994); Fondevilla et al. (2016) |
| *F.* *microtuberculata** | Aix-en-Provence (upper Campanian) | La Cairanne, Aix-en-Provence, France | 160 | 1.84–2.52 | 190–600 (300) | ? | ? | Garcia and Vianey-Liaud (2001); Vianey-Liaud et al. (2003) |
| *F.* *mohabeyi** | Lameta Formation (Maastrichtian) | Dohad of Panchmahals and Phensani, Balasinor, Sonipur and Waniawao of Kheda, Gujarat; Dholiya, Dholiya Raipuriya and Padlya, Dhar, Madhya Pradesh, India | 160–190; 121–158 | 1.80–1.90; 1.65–1.90; 0.90–1.80 | 400–800 (575) | 3.00:1; 3.06:1; 3.10:1; 2.00–3.70:1 (2.95:1) | 140–210; 200–500 (300) | Mohabey and Mathur (1989); Khosla and Sahni (1995); Mohabey (1998); Dhiman et al. (2023) |
| *F*. *padiyalensis** | Lameta Formation (Maastrichtian) | Padiyal, Akhada, Dholiya Raipuriya, and Jhaba, Dhar, Madhya Pradesh, India | 154–168 | 1.12–1.68; 1.70–2.80 | 600–700 (667) | 3.95:1; 2.70–4.60:1 (3.60:1) | 70–210; 300–500 (400) | Khosla and Sahni (1995); Dhiman et al. (2023) |
| *F. petralta** | Aix-en-Provence (lower maastrichtian); Aren Formation (Upper Cretaceous); Tremp Formation (Maastrichtian) | Roquehautes-Grand Creux B-D, Aix-en-Provence, France; Fontllonga 6 and Moro, Tremp Basin, Southern Pyrenees, Lleida, Spain | ? | 1.52; 1.06–1.94 (1.56); 1.60 | 400–600; 270–970 (580) | ? | ? | Vianey-Liaud et al. (1994); Vianey-Liaud and Lopez-Martinez (1997) |
| *F*. *roussentensis* | Aix-en-Provence (Maastrichtian) | Rousset-Village, Roquehautes-Crete du Marbre, Villeveyrac Basin, and Argelliers-Montarnaud, Aix-en-Provence, France | 190; 180x150 (deformed) | 1.49-1.77; 1.11-1.64 (1.29) | 1500 | 2.70:1 | ? | Vianey-Liaud et al. (1994); Sellés et al. (2013) |
| *Litosoolithus poyosi* | Villalba de la Sierra Formation (Campanian-Maastrichtian) | Poyos fossil site, Guadalajara Province and Zafra de Zancara, Cuenca Province, Castilla-La Mancha, Spain | 220–254 (243) x 182–199 (190) | 0.80–1.40 (1.01) | 120–1030 (470) | 2.60:1–4.00:1 | ? | Sanguino et al. (2025) |

**Table S2**. Lithology of in-situ sauropod eggs, clutches and nesting grounds (Faveoloolithidae, Fusioolithidae, and Megaloolithidae). Abbreviation: NA, not available.

| Ootaxon | Material | Lithology | Pedogenetic features | Formation | Locality | Lithology references | Remarks |
| --- | --- | --- | --- | --- | --- | --- | --- |
| Faveoloolithidae | | | | | | | |
| *Faveoloolithus ningxiaensis* | Clutch(es) | Coarse |  | NA | Bayinwulashan cahanaobao, Alxa Left Banner (Inner Mongolia), Ningxia, China | Zhao and Ding (1976); Zhang (2010) |  |
| *Faveoloolithus* sp. | Clutch(es) | Fine | Yes | Seonso | Bosung County, Chullanam-do, South Korea | Huh and Zelenitsky (2002); Kim et al. (2009); Paik et al. (2004) |  |
| *Hemifaveoloolithus muyushanensis* | Clutch(es) | Coarse |  | Chichengshan | Tiantai, Zhejiang, China | Wang et al. (2011, 2012b) |  |
| *Parafaveoloolithus macroporus* | Clutch(es) | Fine |  | Laijia | Laijia Village, Tiantai, Zhejiang, China | Zhang (2010) |  |
| *Parafeveoloolithus fengguangcunensis* | Clutch(es) | Coarse |  | Dongyuan | Heyuan Basin, Guangdong, China | Fang et al. (2005) | Originally *Dendroolithus fengguangcunensis*. |
| *Parafaveoloolithus xixiaensis* | Clutch(es) | Fine |  | Zhaoying | Danshui Town, Xixia, Henan, China | He et al. (2025) |  |
| *Parafaveoloolithus* sp. | Clutch(es) | Coarse |  | Barun Goyot | Ologoy-Ulan-Tsav, Gobi, Mongolia | Sochava (1969); Mikhailov (1994b) | Originally *Faveoloolithus ningxiaensis*. |
| *Propagoolithus widoensis* | Colonial nesting ground (one of 9 horizons) | Fine | Yes | Wido Volcanics | Wi Island, Buan County, North Jeolla Province, South Korea | Kim et al. (2019, 2021) |  |
| *Propagoolithus widoensis* | Colonial nesting ground (one of 9 horizons) | Fine | Yes | Wido Volcanics | Wi Island, Buan County, North Jeolla Province, South Korea | Kim et al. (2019, 2021) |  |
| *Propagoolithus widoensis* | Colonial nesting ground (one of 9 horizons) | Fine | Yes | Wido Volcanics | Wi Island, Buan County, North Jeolla Province, South Korea | Kim et al. (2019, 2021) |  |
| *Propagoolithus widoensis* | Colonial nesting ground (one of 9 horizons) | Fine | Yes | Wido Volcanics | Wi Island, Buan County, North Jeolla Province, South Korea | Kim et al. (2019, 2021) |  |
| *Propagoolithus widoensis* | Colonial nesting ground (one of 9 horizons) | Fine | Yes | Wido Volcanics | Wi Island, Buan County, North Jeolla Province, South Korea | Kim et al. (2019, 2021) |  |
| *Propagoolithus widoensis* | Colonial nesting ground (one of 9 horizons) | Fine | Yes | Wido Volcanics | Wi Island, Buan County, North Jeolla Province, South Korea | Kim et al. (2019, 2021) |  |
| *Propagoolithus widoensis* | Colonial nesting ground (one of 9 horizons) | Fine | Yes | Wido Volcanics | Wi Island, Buan County, North Jeolla Province, South Korea | Kim et al. (2019, 2021) |  |
| *Propagoolithus widoensis* | Colonial nesting ground (one of 9 horizons) | Fine | Yes | Wido Volcanics | Wi Island, Buan County, North Jeolla Province, South Korea | Kim et al. (2019, 2021) |  |
| *Propagoolithus widoensis* | Colonial nesting ground (one of 9 horizons) | Fine | Yes | Wido Volcanics | Wi Island, Buan County, North Jeolla Province, South Korea | Kim et al. (2019, 2021) |  |
| Faveoloolithidae indet. | Clutch(es) | Coarse |  | Sihwa | Sihwa Bay, Whaseong, Gyeonggi Province, South Korea | Lee (2003) |  |
| Faveoloolithidae indet. | Clutch(es) (Locations 1-1 I5, 1-2, 1-4) | Fine |  | Sihwa | Hanyom, SihwaBasin, South Korea | Kim et al. (2009) |  |
| Faveoloolithidae indet. | Clutch(es) (Location 3-1 Islets between) | Fine |  | Sihwa | Hanyom, Sihwa Basin, South Korea | Kim et al. (2009) |  |
| Faveoloolithidae indet. | Clutch(es) (Locations 3-2 HH and JH, 3-3, and 3-4) | Coarse |  | Sihwa | Hanyom, Sihwa Basin, South Korea | Kim et al. (2009) |  |
| Faveoloolithidae indet. | Clutch(es) (Location 3-5) | Coarse |  | Sihwa | Hanyom, Sihwa Basin, South Korea | Kim et al. (2009) |  |
| Faveoloolithidae indet. | Clutch(es) (Location 3-6) | Coarse |  | Sihwa | Hanyom, Sihwa Basin, South Korea | Kim et al. (2009) |  |
| Faveoloolithidae indet. | Clutch(es) (Locations 4-1 JH, 4-2, 4-3, and 4-4) | Coarse |  | Sihwa | Hanyom, Sihwa Basin, South Korea | Kim et al. (2009) |  |
| Faveoloolithidae indet. | Clutch(es) (Location 4-5) | Coarse |  | Sihwa | Hanyom, Sihwa Basin, South Korea | Kim et al. (2009) |  |
| Faveoloolithidae indet. | Clutch(es) (Location 5-2) | Coarse |  | Sihwa | Hanyom, Sihwa Basin, South Korea | Kim et al. (2009) |  |
| Faveoloolithidae indet. | Clutch(es) (Location 5-3) | Coarse |  | Sihwa | Hanyom, Sihwa Basin, South Korea | Kim et al. (2009) |  |
| Faveoloolithidae indet. | Clutch(es) (Location 5-4) | Coarse |  | Sihwa | Hanyom, Sihwa Basin, South Korea | Kim et al. (2009) |  |
| Faveoloolithidae indet. | Clutch(es) (Locations 6-1 H and 6-2) | Coarse |  | Sihwa | Hanyom, Sihwa Basin, South Korea | Kim et al. (2009) |  |
| Faveoloolithidae indet. | Clutch(es) (Locations 6-3 and 6-4) | Coarse |  | Sihwa | Hanyom, Sihwa Basin, South Korea | Kim et al. (2009) |  |
| Faveoloolithidae indet. | Clutch(es) (Location 6-5) | Coarse |  | Sihwa | Hanyom, Sihwa Basin, South Korea | Kim et al. (2009) |  |
| Faveoloolithidae indet. | Clutch(es) (Location 6-6) | Coarse |  | Sihwa | Hanyom, Sihwa Basin, South Korea | Kim et al. (2009) |  |
| Faveoloolithidae indet. | Clutch(es) (Location 6-7) | Coarse |  | Sihwa | Hanyom, Sihwa Basin, South Korea | Kim et al. (2009) |  |
| Faveoloolithidae indet. | Clutch(es) (Location 6-8) | Coarse |  | Sihwa | Hanyom, Sihwa Basin, South Korea | Kim et al. (2009) |  |
| Faveoloolithidae indet. | Clutch(es) (Location 6-9) | Coarse |  | Sihwa | Hanyom, Sihwa Basin, South Korea | Kim et al. (2009) |  |
| Faveoloolithidae indet. | Clutch(es) (Location 9-1 D) | Coarse |  | Sihwa | Hanyom, Sihwa Basin, South Korea | Kim et al. (2009) |  |
| Faveoloolithidae indet. | Clutch(es) (Location 9-2) | Coarse |  | Sihwa | Hanyom, Sihwa Basin, South Korea | Kim et al. (2009) |  |
| Faveoloolithidae indet. | Clutch(es) (Location 9-3) | Coarse |  | Sihwa | Hanyom, Sihwa Basin, South Korea | Kim et al. (2009) |  |
| Faveoloolithidae indet. | Clutch(es) (Location 9-4) | Coarse |  | Sihwa | Hanyom, Sihwa Basin, South Korea | Kim et al. (2009) |  |
| Faveoloolithidae indet. | Clutch(es) | Coarse |  | Haman | Coast of Sinsudo Island, Samcheonpo, Korea | Paik et al. (2012) |  |
| Faveoloolithidae indet. | Clutch(es) | Coarse |  | NA | Algui Ulan Tsav, Gobi, Mongolia | Watabe et al. (2010) |  |
| Faveoloolithidae indet. | Colonial nesting ground (one of 5 horizons) | Coarse | Yes | Los Llanos | Sanagasta, La Rioja, Argentina | Grellet-Tinner and Fiorelli (2010); Fiorelli et al. (2012) |  |
| Faveoloolithidae indet. | Colonial nesting ground (one of 5 horizons) | Coarse | Yes | Los Llanos | Sanagasta, La Rioja, Argentina | Grellet-Tinner and Fiorelli (2010); Fiorelli et al. (2012) |  |
| Faveoloolithidae indet. | Colonial nesting ground (one of 5 horizons) | Coarse | Yes | Los Llanos | Sanagasta, La Rioja, Argentina | Grellet-Tinner and Fiorelli (2010); Fiorelli et al. (2012) |  |
| Faveoloolithidae indet. | Colonial nesting ground (one of 5 horizons) | Coarse | Yes | Los Llanos | Sanagasta, La Rioja, Argentina | Grellet-Tinner and Fiorelli (2010); Fiorelli et al. (2012) |  |
| Faveoloolithidae indet. | Colonial nesting ground (one of 5 horizons) | Coarse | Yes | Los Llanos | Sanagasta, La Rioja, Argentina | Grellet-Tinner and Fiorelli (2010); Fiorelli et al. (2012) |  |
| Faveoloolithidae indet. | Clutch(es) (Egg bed 3 of Salitral Ojo de Agua) | Coarse | Yes | Allen | Arriagada I, Salitral Ojo de Agua, Rio Negro, Argentina | Salgado et al. (2007) |  |
| Faveoloolithidae indet. | Clutch(es) (Egg bed 2 of Salitral de Santa Rosa-Salinas de Trapalco) | Coarse | Yes | Allen | Santos I, Salitral de Santa Rosa-Salinas de Trapalco, Rio Negro, Argentina | Salgado et al. (2007) |  |
| Faveoloolithidae indet. | Clutch(es) (Egg bed 3 of Salitral de Santa Rosa-Salinas de Trapalco) | Coarse | Yes | Allen | Santos II and Berthe IV, Salitral de Santa Rosa-Salinas de Trapalco, Rio Negro, Argentina | Salgado et al. (2007) |  |
| Fusioolithidae | | | | | | | |
| *Fusioolithus baghenisis* | Clutch(es) | Coarse | Yes | Lameta | Balasinor Quarry, Jetholi and Dhuvadiya, Kheda, Gujarat, India | Mohabey (1998) | Originally *Megaloolithus balasinorensis* (Vianey-Liaud et al., 2003; Fernández and Khosla, 2015). |
| *Fusioolithus baghensis* | Clutch(es) | Fine | Yes | Villalba de la Sierra | Poyos fossil site, Guadalajara Province, Castilla-La Mancha, Spain | Sanguino et al. (2025) |  |
| *Fusioolithus* *dughii* | Clutch(es) | Fine |  | NA | Commune de Rousset, near Cairanne, Aix-en-Provence, France | Williams et al. (1984) | Originally *Cairanoolithus* (Fernández and Khosla, 2015). |
| *Fusioolithus mammillare* | Clutch(es) (one of multiple stratigraphic levels) | Fine |  | NA | Commune de Rousset, Aix-en-Provence, France | Williams et al. (1984) | Originally *Megaloolithus* (Fernández and Khosla, 2015). |
| *Fusioolithus mammillare* | Clutch(es) (one of multiple stratigraphic levels) | Fine |  | NA | Commune de Chateauneuf Le Rouge, Aix-en-Provence, France | Williams et al. (1984) | Originally *Megaloolithus* (Fernández and Khosla, 2015). |
| *Fusioolithus microtuberculata* | Clutch(es) | Fine |  | NA | La Cairanne, Aix-en-Provence, France | Garcia and Vianey-Liaud (2001); Vianey-Liaud et al. (2003) | Originally *Megaloolithus* (Fernández and Khosla, 2015). |
| *Fusioolithus* *mohabeyi* | Clutch(es) | Coarse | Yes | Lameta | Phensani, Balasinor, Sonipur and Waniawao, Kheda, Gujarat, India | Mohabey (1998) | Originally *Megaloolithus phensaniensis* (Vianey-Liaud et al., 2003; Fernández and Khosla, 2015). |
| *Fusioolithus* cf. *roussentensis* | Clutch(es) | Fine |  | Tremp | Coll de Nargo, Catalunya, Lleida, Spain | Sellés et al. (2013) | Originally *Cairanoolithus* (Fernández and Khosla, 2015). |
| *Litosoolithus poyosi* | Clutch(es) | Fine | Yes | Villalba de la Sierra | Poyos fossil site, Guadalajara Province, Castilla-La Mancha, Spain | Sanguino et al. (2025) |  |
| Megaloolithidae | | | | | | | |
| *Megaloolithus cylindricus* | Clutch(es) | Coarse | Yes | Lameta | Rahioli, Kheda, Gujarat, India | Mohabey (1998) | Including *Megaloolithus rahioliensis*. |
| *Megaloolithus dhoridungriensis* | Clutch(es), associated with a juvenile sauropod. | Coarse | Yes | Lameta | Dhoridungri, Gujarat, India | Mohabey (1998) |  |
| *Megaloolithus dhoridungriensis* | Clutch(es), associated with a sauropod hatchling. | Coarse | Yes | Lameta | Near Dholi Dungri, Gujarat, western India | Wilson et al. (2010) |  |
| *Megaloolithus jabalpurensis* | Clutch(es) (Site B) | Coarse | Yes | Lameta | Pavna, Chandrapur, Maharashtra, India | Mohabey (1996, 1998) | Originally *Megaloolithus matleyi*. |
| *Megaloolithus jabalpurensis* | Nesting ground (Site A) | Coarse | Yes | Lameta | Pavna, Chandrapur, Maharashtra, India | Mohabey (1996) | Originally *Megaloolithus matleyi*. |
| *Megaloolithus jabalpurensis* | Colonial nesting ground (Egg bed 3) with embryonic bones | Fine |  | Anacleto | Auca Mahuevo, Neuquén Province, Patagonia, Argentina | Chiappe et al. (1998, 2001); Grellet-Tinner et al. (2004); Jackson et al. (2013) | Originally *Megaloolithus patagonicus*. Eggs of Titanosauria. |
| *Megaloolithus jabalpurensis* | Colonial nesting ground (Egg bed 4) | Fine |  | Anacleto | Auca Mahuevo, Neuquén Province, Patagonia, Argentina | Chiappe et al. (2001, 2004) | Originally *Megaloolithus patagonicus*. |
| *Megaloolithus jabalpurensis* | Colonial nesting ground (Egg bed 2) | Fine |  | Anacleto | Auca Mahuevo, Neuquén Province, Patagonia, Argentina | Chiappe et al. (2001, 2004) | Originally *Megaloolithus patagonicus*. |
| *Megaloolithus jabalpurensis* | Colonial nesting ground (Egg bed 1) | Fine |  | Anacleto | Auca Mahuevo, Neuquén Province, Patagonia, Argentina | Chiappe et al. (2001, 2004) | Originally *Megaloolithus patagonicus*. |
| *Megaloolithus jabalpurensis* | Clutch(es) | Fine |  | Bajo de la Carpa | Gran Neuquén neighbourhood, Neuquén, Neuquén Province, Patagonia, Argentina | Calvo et al. (1997) | Originally *Megaloolithus patagonicus*. |
| *Megaloolithus jabalpurensis* (Type 2A) | Clutch(es) (Egg level 3 of Salitral de Santa Rosa-Salinas de Trapalco) | Coarse | Yes | Allen | Berthe IV, Salitral de Santa Rosa-Salinas de Trapalco, Rio Negro, Argentina | Salgado et al. (2007, 2009) | Originally *Megaloolithus* sp. |
| *Megaloolithus khempurensis* | Clutch(es) | Coarse | Yes | Lameta | Khempur and Werasa, Kheda, Gujarat, India | Mohabey (1998) | *Megaloolithus walpurensis*? (Vianey-Liaud et al., 2003) |
| *Megaloolithus megadermus* | Clutch(es) | Coarse | Yes | Lameta | Dholidhanti and Paori, Dohad, Panchmahals, India | Mohabey (1998) |  |
| *Megaloolithus siruguei* | Clutch(es) (Egg horizon 1) | Fine | Yes | Tremp | Coll de Nargo, Lleida, Catalonia, Spain | Jackson (2007); Vila et al. (2010) |  |
| *Megaloolithus siruguei* | Clutch(es) (Egg horizon 3) | Fine | Yes | Tremp | Coll de Nargo, Lleida, Catalonia, Spain | Jackson (2007); Vila et al. (2010) |  |
| *Megaloolithus siruguei* | Clutch(es) (Egg horizon 5) | Fine | Yes | Tremp | Coll de Nargo, Lleida, Catalonia, Spain | Jackson (2007); Vila et al. (2010) |  |
| *Megaloolithus siruguei* | Clutch(es) (Egg horizon 6) | Coarse | Yes | Tremp | Coll de Nargo, Lleida, Catalonia, Spain | Jackson (2007); Vila et al. (2010) |  |
| *Megaloolithus siruguei* | Clutch(es) (Egg horizon 7) | Coarse | Yes | Tremp | Coll de Nargo, Lleida, Catalonia, Spain | Jackson (2007); Vila et al. (2010) |  |
| *Megaloolithus siruguei* | Clutch(es) (Egg horizon 8) | Coarse | Yes | Tremp | Coll de Nargo, Lleida, Catalonia, Spain | Jackson (2007); Vila et al. (2010) |  |
| *Megaloolithus siruguei* | Clutch(es) (Facies E) | Fine | Yes | Tremp | Font del Bullidor, Vallcebre Syncline, Spain | Vila et al. (2010) |  |
| *Megaloolithus siruguei* | Clutch(es) (one of multiple stratigraphic levels) | Fine | Yes | Tremp | Coll de Nargo, Catalunya, Lleida, Spain | Sellés et al. (2013) |  |
| *Megaloolithus siruguei* | Clutch(es) (one of multiple stratigraphic levels) | Fine | Yes | Tremp | Coll de Nargo, Catalunya, Lleida, Spain | Sellés et al. (2013) |  |
| *Megaloolithus siruguei* | Clutch(es) (one of multiple stratigraphic levels) | Fine | Yes | Tremp | Coll de Nargo, Catalunya, Lleida, Spain | Sellés et al. (2013) |  |
| *Megaloolithus siruguei* | Clutch(es) (one of multiple stratigraphic levels) | Fine | Yes | Tremp | Coll de Nargo, Catalunya, Lleida, Spain | Sellés et al. (2013) |  |
| *Megaloolithus siruguei* | Clutch(es) (one of multiple stratigraphic levels) | Fine | Yes | Tremp | Coll de Nargo, Catalunya, Lleida, Spain | Sellés et al. (2013) |  |
| *Megaloolithus siruguei* | Clutch(es) (one of multiple stratigraphic levels) | Fine | Yes | Tremp | Coll de Nargo, Catalunya, Lleida, Spain | Sellés et al. (2013) |  |
| *Megaloolithus siruguei* | Clutch(es) (one of multiple stratigraphic levels) | Coarse |  | Tremp | Coll de Nargo, Catalunya, Lleida, Spain | Sellés et al. (2013) |  |
| *Megaloolithus siruguei* | Clutch(es) (one of multiple stratigraphic levels) | Fine | Yes | Tremp | Coll de Nargo, Catalunya, Lleida, Spain | Sellés et al. (2013) |  |
| *Megaloolithus siruguei* | Clutch(es) (one of multiple stratigraphic levels) | Fine | Yes | Tremp | Coll de Nargo, Catalunya, Lleida, Spain | Sellés et al. (2013) |  |
| *Megaloolithus siruguei* | Clutch(es) (one of multiple stratigraphic levels) | Fine | Yes | Tremp | Coll de Nargo, Catalunya, Lleida, Spain | Sellés et al. (2013) |  |
| *Megaloolithus siruguei* | Clutch(es) (one of multiple stratigraphic levels) | Fine | Yes | Tremp | Coll de Nargo, Catalunya, Lleida, Spain | Sellés et al. (2013) |  |
| *Megaloolithus siruguei* | Clutch(es) (one of multiple stratigraphic levels) | Coarse |  | Tremp | Coll de Nargo, Catalunya, Lleida, Spain | Sellés et al. (2013) |  |
| *Megaloolithus siruguei* | Clutch(es) | Fine | Yes | Tremp | Biscarri, Isona, Lleida, Spain | Lopez-Martinez et al. (2000) |  |
| *Megaloolithus siruguei* | Clutch(es) (C1 nesting horizon) | Fine | Yes | Densus-Ciula | Tustea, Hateg Basin, Romania | Grigorescu et al. (1994, 2010); Grigorescu (2016); Botfalvai et al. (2017) |  |
| *Megaloolithus siruguei* | Clutch(es) (C2 nesting horizon) | Fine | Yes | Densus-Ciula | Tustea, Hateg Basin, Romania | Grigorescu et al. (1994, 2010); Grigorescu (2016); Botfalvai et al. (2017) |  |
| *Megaloolithus* cf. *siruguei* | Clutch(es) (Horizon E) | Fine | Yes | Sanpetru | Totesti-baraj, Hateg Basin, Romania | Codrea et al. (2002); Grigorescu et al. (1994, 2010); Grellet-Tinner et al. (2012) |  |
| *Megaloolithus* cf. *siruguei* | Clutch(es) (Horizon A) | Fine | Yes | Sanpetru | Totesti-baraj, Hateg Basin, Romania | Codrea et al. (2002); Grigorescu et al. (1994, 2010); Grellet-Tinner et al. (2012) |  |
| *Megaloolithus* cf. *siruguei* | Clutch(es) (Horizon K) | Fine | Yes | Sanpetru | Totesti-baraj, Hateg Basin, Romania | Codrea et al. (2002); Grigorescu et al. (1994, 2010); Grellet-Tinner et al. (2012) |  |
| *Megaloolithus* cf. *siruguei* | Clutch(es) (Horizon B) | Fine | Yes | Sanpetru | Totesti-baraj, Hateg Basin, Romania | Codrea et al. (2002); Grigorescu et al. (1994, 2010); Grellet-Tinner et al. (2012) |  |
| *Megaloolithus* cf. *siruguei* | Clutch(es) (Horizons G and H) | Fine | Yes | Sanpetru | Totesti-baraj, Hateg Basin, Romania | Codrea et al. (2002); Grigorescu et al. (1994, 2010); Grellet-Tinner et al. (2012) |  |
| *Megaloolithus* cf. *siruguei* | Clutch(es) (Find8) | Fine | Yes | Sanpetru | Nalat-Vad, Hateg Basin, Romania | Smith et al. (2002); Grigorescu et al. (1994, 2010) |  |
| *Megaloolithus* cf. *siruguei* | Clutch(es) (Find9) | Fine | Yes | Sanpetru | Nalat-Vad, Hateg Basin, Romania | Smith et al. (2002); Grigorescu et al. (1994, 2010) |  |
| *Megaloolithus* cf. *siruguei* | Clutch(es) (Find2) | Fine | Yes | Sanpetru | Nalat-Vad, Hateg Basin, Romania | Smith et al. (2002); Grigorescu et al. (1994, 2010) |  |
| *Megaloolithus* cf. *siruguei* | Clutch(es) (Find6) | Fine |  | Sanpetru | Nalat-Vad, Hateg Basin, Romania | Smith et al. (2002); Grigorescu et al. (1994, 2010) |  |
| *Megaloolithus* cf. *siruguei* | Clutch(es) (Find7) | Coarse | Yes | Sanpetru | Nalat-Vad, Hateg Basin, Romania | Smith et al. (2002); Grigorescu et al. (1994, 2010) |  |
| *Megaloolithus* sp. | Clutch(es) | Coarse | Yes | Lameta | Salbardi, Amravati, Maharashtra and Betul, India | Srivastava and Mankar (2015) |  |
| *Megaloolithus* sp. | Colonial nesting ground | Coarse | Yes | Aren | Barranc de la Costa Gran, Bastus, Lleida, Spain | Sanz et al. (1995); Sander et al. (1998); Moratalla et al. (1995); Diaz-Molina et al. (2007) |  |
| *Megaloolithus* sp. | Clutch(es) | Fine | Yes | Densus-Ciula | Tustea, Hateg Basin, Romania | Venczel et al. (2015) |  |
| *Megaloolithus* sp. | Clutch(es) | Coarse | Yes | Lameta | Bagh-Kukshi, Dhar, Madhya Pradesh and Rahioli/Raiyoli, Kheda, Gujarat, India | Dhiman et al. (2021) |  |
| Megaloolithidae indet. | Clutch(es) | Coarse | Yes | Allen | Bajo de Arriagada, Patagonia, Rio Negro, Argentina | Genise and Sarzetti (2011) |  |
| Megaloolithidae indet. (Type 2B) | Clutch(es) (Egg level 2 of Salitral Ojo de Agua) | Coarse |  | Allen | Salitral Ojo de Agua, Rio Negro, Argentina | Salgado et al. (2007) |  |
| Eggs of "*Hypselosaurus priscus*" | Colonial nesting ground | Fine |  | NA (Marines Rouges Inferieures) | Rennes-le-chateu, Aude, France | Cousin et al. (1989) |  |
| Titanosaur eggs | Colonial nesting horizon (one of two horizons) | Coarse | Yes | Serra da Galga Formation | Ponte Alta, Uberaba Municipality, Minas Gerais State, Brazil | Fiorelli et al. (2022) |  |
| Titanosaur eggs | Colonial nesting horizon (one of two horizons) | Coarse | Yes | Serra da Galga Formation | Ponte Alta, Uberaba Municipality, Minas Gerais State, Brazil | Fiorelli et al. (2022) |  |
| Titanosaur eggs | Clutch(es) (Level 1) | Fine | Yes | Ciénaga del Río Huaco | Quebrada de Santo Domingo, La Rioja, Argentina | Hechenleitner et al. (2020) |  |
| Titanosaur eggs | Clutch(es) (Level 2) | Fine | Yes | Ciénaga del Río Huaco | Quebrada de Santo Domingo, La Rioja, Argentina | Hechenleitner et al. (2020) |  |
| Titanosaur eggs | Clutch(es) (Level 3) | Fine | Yes | Ciénaga del Río Huaco | Quebrada de Santo Domingo, La Rioja, Argentina | Hechenleitner et al. (2020) |  |

**Table S3**. Approximate paleolatitudes of sauropod egg localities. Abbreviations: Fa, faveoloolithidae; Fu, Fusioolithidae; M, Megaloolithidae. *The eggshell was classified here into Faveoloolithidae based on Figure 2 of Powell (1987).

| Oofamily type | Formation | Locality | Approximate age (Ma) | Approximate paleolatitude (°) | References |
| --- | --- | --- | --- | --- | --- |
| Fa | Lijiacun | Yangyuhe, Shangluo, Shaanxi, China | 80 | 39.1 | Zheng et al. (2018) |
| Fa | Gaogou | Xichuan Basin, Henan, China | 91.5 | 34.4 | Jin (2013) |
| Fa | Zhaoying | Xixia Basin, Henan, China | 86 | 35.7 | He et al. (2025) |
| Fa | Zoumagang | Diangou, Yangcheng, Xixia, Henan, China | 91.5 | 34.6 | Fang et al. (1998) |
| Fa | Gaogou | Qinglongshan, Yunxian, Hubei, China | 90 | 37.7 | Zhou et al. (1998) |
| Fa | Chaochuan | Yongkang Basin, Zhejiang, China | 94 | 30.7 | Zhou et al. (2010); Jin (2013) |
| Fa | Jinhua | Jinqu Basin, Zhejiang, China | 91.5 | 32.1 | Jin (2013) |
| Fa | Laijia and Chichengshan | Tiantai, Zhejiang, China | 95 | 36.4 | Fang et al. (2000); Zhang (2010); Jin (2013); Wang et al. (2011, 2012) |
| Fa | Zhoutian | Pingxiang Basin, Jiagxi, China | 89 | 30.4 | Zou et al. (2013) |
| Fa | Dongyuan | Heyuan Basin, Guangdong, China | 65 | 29.1 | Fang et al. (2005) |
| Fa | Barun Goyot | Gilbent, Mongolia | 75 | 45.6 | Mikhailov (1994) |
| Fa | Barun Goyot | Khulsan, Mongolia | 75 | 45.4 | Mikhailov (1994) |
| Fa | Barun Goyot | Ikh Shunkht, Mongolia | 75 | 46.5 | Mikhailov (1994); Carpenter and Alf (1994) |
| Fa | Barun Goyot | Khermeen Tsav I and II, Mongolia | 75 | 45.0 | Mikhailov (1994); Carpenter and Alf (1994) |
| Fa | Barun Goyot | Ologoy-Ulan-Tsav, Gobi, Mongolia | 75 | 47.1 | Sochava (1969); Mikhailov (1994) |
| Fa | Bayn Shiree | Bayn Shiree, Mongolia | 91.5 | 44.8 | Ariunchimeg (2000) |
| Fa | Djadokhta | Bayn Dzak, Mongolia | 73 | 46.4 | Mikhailov (1994) |
| Fa | Khuren Dukh | Khuren-Dukh, Mongolia | 104 | 46.4 | Mikhailov (1994) |
| Fa | NA | Algui Ulan Tsav, Gobi, Mongolia | 124.75 | 45.5 | Ariunchimeg (2000); Watabe et al. (2010) |
| Fa | NA | Nogon Tsav, Mongolia | 69 | 44.9 | Watabe et al. (2010) |
| Fa | Haman | Sinsudo Island, Samcheonpo, Korea | 100 | 43.2 | Paik et al. (2012) |
| Fa | Seonso | Bosung, Chullanam, South Korea | 80 | 41.0 | Huh and Zelenitsky (2002) |
| Fa | Sihwa | Hanyom, Sihwa Basin, South Korea | 110 | 41.3 | Lee (2003); Kim et al. (2009, 2025) |
| Fa | Wido Volcanics | Wi Island, Buan, North Jeolla, Southe Korea | 87 | 42.1 | Kim et al. (2019, 2022) |
| Fa | Asencio | Colonia, 12a. Police Section, stream Miguelete Tala, Uruguay | 95 | -38.8 | Mones (1980) |
| Fa | Guichon | Quebracho, Paysandu, Uruguay | 100 | -36.1 | Soto et al. (2012) |
| Fa | Allen | Santos II and IV, Río Negro, Argentina | 74 | -45.7 | Salgado et al. (2007); Fernández (2013); Fernández and Khosla (2015) |
| Fa | Allen | Cerro Bonaparte and Berthe I, III and IV, Río Negro, Argentina | 74 | -46.1 | Salgado et al. (2007); Fernández (2013); Fernández and Khosla (2015) |
| Fa | Allen | Salitral de Santa Rosa, Río Negro, Argentina | 74 | -45.7 | Salgado et al. (2007); Fernández (2013); Fernández and Khosla (2015) |
| Fa | Allen | Salitral Ojo de Agua, Río Negro, Argentina | 74 | -45.4 | Salgado et al. (2007); Fernández (2013); Fernández and Khosla (2015) |
| Fa | Los Llanos | Sanagasta, La Rioja, Argentina | 125 | -26.1 | Grellet-Tinner and Fiorelli (2010); Fiorelli et al. (2012) |
| Fa* | Los Alamitos | Los Alamitos, Rio Negro Province, Argentina | 74 | -47.7 | Powell (1987) |
| Fu | NA | Clos-La-Neuve, Roquehautes-Grand Creux-A, B, and D, Roquehautes-Crete du Marbre, Villeveyrac Basin, La Cairanne, and Argelliers-Montarnaud, and Saint-Chinian, Aix-en-Provence, France | 75 | 33.2 | Vianey-Liaud et al. (1994, 2003); Garcia and Vianey-Liaud (2001) |
| Fu | Maurine/NA | Rousset-Erben, La Cardeline, Les Breguieres, Commune de Rousset, Commune de Chateauneuf Le Rouge, and Rousset-Village, Aix-en-Provence, France; Roque Fumade, Upper Aude Valley, France | 65 | 32.5 | Williams et al. (1984): Vianey-Liaud et al. (1994); Fondevilla et al. (2016) |
| Fu | Maurine | Les Boudous and Gourg de l'Encantado, Upper Aude Valley, France | 65 | 32.0 | Fondevilla et al. (2016) |
| Fu | Tremp | Coll de Nargo, Lleida, Catalonia, Spain | 65 | 31.0 | Sellés et al. (2013) |
| Fu | Tremp | Fontllonga 6 and Bastus, Lleida, Catalonia, Spain | 65 | 29.5 | Vianey-Liaud and Lopez-Martinez (1997) |
| Fu | Tremp | Abella, Lleida, Catalonia, Spain | 65 | 31.0 | Vianey-Liaud and Lopez-Martinez (1997) |
| Fu | Tremp | Suterranya, Lleida, Catalonia, Spain | 65 | 29.6 | Vianey-Liaud and Lopez-Martinez (1997) |
| Fu | Villalba de la Sierra | Poyos fossil site, Guadalajara Province, Castilla-La Mancha, Spain | 73 | 27.2 | Sanguino et al. (2025) |
| Fu | Villalba de la Sierra | Zafra de Zancara, Cuenca Province, Castilla-La, Mancha, Spain | 73 | 26.6 | Sanguino et al. (2025) |
| Fu | Aren | Moro, Lleida, Catalonia, Spain | 70 | 30.0 | Vianey-Liaud and Lopez-Martinez (1997) |
| Fu | Lameta | Rahioli, Kheda, Gujarat, India | 67.5 | -20.4 | Mohabey (1984, 1998); Srivastava et al. (1986) |
| Fu | Lameta | Balasinor Taluka, Kheda, Gujarat, India | 67.5 | -20.5 | Mohabey (1984, 1998); Srivastava et al. (1986) |
| Fu | Lameta | Jetholi, Kheda, Gujarat, India | 67.5 | -20.4 | Mohabey (1984, 1998); Srivastava et al. (1986) |
| Fu | Lameta | Dhuvadiya, Kheda, Gujarat, India | 67.5 | -20.5 | Mohabey (1984, 1998); Srivastava et al. (1986) |
| Fu | Lameta | Phensani, Kheda, Gujarat, India | 67.5 | -20.5 | Mohabey (1984, 1998); Srivastava et al. (1986) |
| Fu | Lameta | Sonipur, Kheda, Gujarat, India | 67.5 | -20.6 | Mohabey (1984, 1998); Srivastava et al. (1986) |
| Fu | Lameta | Waniawao, Kheda, Gujarat, India | 67.5 | -20.9 | Mohabey (1984, 1998); Srivastava et al. (1986) |
| Fu | Lameta | Dohad, Panchmahals, Gujarat, India | 67.5 | -20.9 | Mohabey and Mathur (1989) |
| Fu | Lameta | Bagh Cave Section, Dhar, Madhya Pradesh, India | 67.5 | -21.7 | Khosla and Sahni (1995); Dhiman et al. (2022, 2023) |
| Fu | Lameta | Pisdura, Dhar, Madhya Pradesh, India | 67.5 | -24.7 | Khosla and Sahni (1995); Dhiman et al. (2022, 2023) |
| Fu | Lameta | Akhada, Dhar, Madhya Pradesh, India | 67.5 | -21.5 | Khosla and Sahni (1995); Dhiman et al. (2022, 2023) |
| Fu | Lameta | Jhaba, Dhar, Madhya Pradesh, India | 67.5 | -21.1 | Khosla and Sahni (1995); Dhiman et al. (2022, 2023) |
| Fu | Lameta | Jamniapura, Dhar, Madhya Pradesh, India | 67.5 | -21.6 | Khosla and Sahni (1995); Dhiman et al. (2022, 2023) |
| Fu | Lameta | Dholiya, Dhar, Madhya Pradesh, India | 67.5 | -21.6 | Khosla and Sahni (1995); Dhiman et al. (2022, 2023) |
| Fu | Lameta | Dholiya Raipuriya, Dhar, Madhya Pradesh, India | 67.5 | -21.6 | Khosla and Sahni (1995); Dhiman et al. (2022, 2023) |
| Fu | Lameta | Padlya, Dhar, Madhya Pradesh, India | 67.5 | -21.7 | Khosla and Sahni (1995); Dhiman et al. (2022, 2023) |
| Fu | Intertrappean beds | Piplanarayanwar, Chhindwara, Madhya Pradesh, India | 67.5 | -23.4 | Lourembam et al. (2022) |
| Fu | Fundo El Triunfo | Laguna Umayo, Bagua Basin, Peru | 75 | -9.4 | Vianey-Liaud et al. (1997, 2003) |
| Fu | Allen | Salitral de Santa Rosa, Salitral Ojo de Agua, and Salitral Moreno, Rio Negro, Argentina | 75 | -44.9 | Simon (2006); Fernández (2013); Fernández and Khosla (2015) |
| Fu | Chorrillo | La Anita Farm, Santa Cruz Province, Patagonia, Argentina | 70 | -55–60 | Chimento et al. (2023); This study |
| M | Lulworth | Durlston Bay, Swanage, Dorset, England | 140 | 33.1 | Ensom (1996) |
| M | NA | Rennes-le-chateu, Aude, France | 65 | 31.8 | Cousin et al. (1989) |
| M | NA | Commune de Fox-Amphoux, Commune de Pourcieux, Commune de Puyloubier, La Begude, Roquehautes, Rousset-Routiers, and Rousset-Village, Aix-en-Provence, France | 65 | 32.5 | Williams et al. (1984); Vianey-Liaud et al. (1994); Garcia (2000) |
| M | NA | Cruzy, Herault, France | 75.6 | 32.1 | Garcia (2000) |
| M | NA | Vitrolles-Couperigne, Bouches-du-Rhone, France | 75.6 | 32.8 | Garcia (2000) |
| M | Densus-Ciula and Sanpetru | Livezi, Nalat-Vad, Tustea, and Totesti-baraj, Hateg Basin, Romania | 68.3 | 32.7 | Grigorescu et al. (1994, 2010); Codrea et al. (2002); Grellet-Tinner et al. (2012); Venczel et al. (2015); Grigorescu (2016) |
| M | Aren | Barranc de la Costa Gran, Bastus, Spain | 70 | 31.4 | Sanz et al. (1995); Peitz (2001); Diaz-Molina et al. (2007) |
| M | Sierra Perenchiza | Iberian Range, Valencia, Spain | 72 | 26.2 | Company (2017) |
| M | Tremp | Coll de Nargo, Lleida, Catalonia, Spain | 65 | 31.0 | Sander et al. (1998); Peitz (2001); Jackson et al. (2008); Vila et al. (2010a); Sellés et al. (2013) |
| M | Tremp | Font del Bullidor, Vallcebre Syncline, Spain | 69 | 30.8 | Vila et al. (2010b) |
| M | Tremp | Biscarri, Isona, Lleida, Spain; Costa de la Coma, Figuerola d'Orcau, Isona I Conca Della, Spain | 65 | 29.6 | Vianey-Liaud and Lopez-Martinez (1997); Lopez-Martinez et al. (2000); Bravo and Gaete (2015) |
| M | Tremp | Gosol, Catalunya, Spain | 65 | 31.1 | Peitz (2001) |
| M | Marilia | Peiropolis and Ponte Alta, Baruru Basin, Marilia, San Paulo, Brazil | 65 | -30.0 | Ribeiro (2002) |
| M | Serra da Galga | Ponte Alta, Uberaba, Minas Gerais, Brazil | 69 | -27.9 | Fiorelli et al. (2022) |
| M | Umayo | Laguna Umayo, Bagua Basin, Peru | 75 | -9.4 | Vianey-Liaud et al. (1997) |
| M | Allen | Arriagada I and III, Salitral Ojo de Agua, Rio Negro, Argentina | 74 | -45.4 | Salgado et al. (2007); Genise and Sarzetti (2011) |
| M | Allen | Cerro Bonaparte and Berthe II-VI and IV, Río Negro Province, Argentina | 74 | -46.1 | Salgado et al. (2007, 2009); Fernández (2013); Fernández and Khosla (2015) |
| M | Allen | Cerro Turtugas, Río Negro Province, Argentina | 74 | -45.6 | Salgado et al. (2007, 2009); Fernández (2013); Fernández and Khosla (2015) |
| M | Allen | Mansilla I and II, Río Negro Province, Argentina | 74 | -45.7 | Salgado et al. (2007, 2009); Fernández (2013); Fernández and Khosla (2015) |
| M | Allen | Garcia I, Río Negro Province, Argentina | 74 | -46.1 | Salgado et al. (2007, 2009); Fernández (2013); Fernández and Khosla (2015) |
| M | Allen | Auca Mahuevo, Neuquén, Patagonia, Argentina | 80 | -43.5 | Chiappe et al. (1998, 2001, 2004); Grellet-Tinner et al. (2004); Jackson et al. (2008) |
| M | Ciénaga del Río Huaco | Quebrada de Santo Domingo, La Rioja, Argentina | 80 | -33.5 | Hechenleitner et al. (2020) |
| M | Bajo de la Carpa | Gran Neuquén, Neuquén, Patagonia, Argentina | 85 | -43.9 | Calvo et al. (1997) |
| M | Lameta | Dhoridungri, Gujarat, India | 67.5 | -20.4 | Mohabey (1998); Wilson et al. (2010) |
| M | Lameta | Rahioli, Kheda, Gujarat, India | 67.5 | -20.4 | Mohabey (1984); Dhiman et al. (2021) |
| M | Lameta | Kevadiya, Kheda, Gujarat, India | 67.5 | -20.3 | Srivastava et al. (1986); Khosla and Sahni (1995); Mohabey (1998) |
| M | Lameta | Khempur, Kheda, Gujarat, India | 67.5 | -20.4 | Srivastava et al. (1986); Khosla and Sahni (1995); Mohabey (1998) |
| M | Lameta | Werasa, Kheda, Gujarat, India | 67.5 | -20.5 | Srivastava et al. (1986); Khosla and Sahni (1995); Mohabey (1998) |
| M | Lameta | Balasinor Taluka, Kheda, Gujarat, India | 67.5 | -20.5 | Srivastava et al. (1986); Khosla and Sahni (1995); Mohabey (1998) |
| M | Lameta | Balasinor, Kheda, Gujarat, India | 67.5 | -20.5 | Srivastava et al. (1986); Khosla and Sahni (1995); Mohabey (1998) |
| M | Lameta | Phensani, Kheda, Gujarat, India | 67.5 | -20.5 | Srivastava et al. (1986); Khosla and Sahni (1995); Mohabey (1998) |
| M | Lameta | Sonipur, Kheda, Gujarat, India | 67.5 | -20.6 | Srivastava et al. (1986); Khosla and Sahni (1995); Mohabey (1998) |
| M | Lameta | Dholidhanti and Paori, Dohad, Panchmahals, Gujarat, India | 67.5 | -20.8 | Mohabey and Mathur (1989); Mohabey (1998) |
| M | Lameta | Walpur, Madhya Pradesh, India | 67.5 | -21.7 | Khosla and Sahni (1995) |
| M | Lameta | Akhada, Dhar, Madhya Pradesh, India | 67.5 | -21.5 | Dhiman et al. (2023) |
| M | Lameta | Jhaba, Dhar, Madhya Pradesh, India | 67.5 | -21.1 | Dhiman et al. (2023) |
| M | Lameta | Padlya, Dhar, Madhya Pradesh, India | 67.5 | -21.7 | Dhiman et al. (2023) |
| M | Lameta | Dholiya Raipuriya, Dhar, Madhya Pradesh, India | 67.5 | -21.6 | Dhiman et al. (2023) |
| M | Lameta | Bagh-Kukshi, Dhar, Madhya Pradesh, India | 67.5 | -21.5 | Dhiman et al. (2021) |
| M | Lameta | Dholiya, Dhar, Madhya Pradesh, India | 67.5 | -21.6 | Khosla and Sahni (1995) |
| M | Lameta | Bagh Cave Section, Padiyal, Madhya Pradesh, India | 67.5 | -21.7 | Khosla and Sahni (1995) |
| M | Lameta | Chui Hill, Jabalpur, Madhya Pradesh, India | 67.5 | -22.2 | Khosla and Sahni (1995); Mohabey (1998) |
| M | Lameta | Pat Baba Mandir, Jabalpur, Madhya Pradesh, India | 67.5 | -22.1 | Khosla and Sahni (1995); Mohabey (1998) |
| M | Lameta | Salbardi, Amravati, Maharashtra, India | 67.5 | -23.3 | Srivastava and Mankar (2015) |
| M | Lameta | Pavna, Chandrapur, Maharashtra, India | 67.5 | -24.9 | Mohabey (1996, 1998) |
| M | Irbzer | Oukdiksou syncline, Morocco | 65 | 20.0 | Garcia et al. (2003) |
| M | Tendrara red beds | Douar Lgara, Tendrara High Plateaus, Morocco | 65 | 19.2 | Chassagne-Manoukian et al. (2013) |
| M | Galula Formation | TZ-07, 20 km west of the town of Mbeya in the Mbeya District, southwestern Tanzania | 105 | -28.7 | Gottfried et al. (2004) |

**References**

Ariunchimeg Y. 2000 Results of studies of dinosaur eggshells (in Abstract of Report Meeting of the Japan-Mongolia Joint Paleontological Expedition). *Hayashibara Mus. Nat. Sci. Res. Bull.* **1**, 131–132.

Botfalvai G, Csiki-Sava Z, Grigorescu D, Vasile S. 2017 Taphonomical and palaeoecological investigation of the Late Cretaceous (Maastrichtian) Tuştea vertebrate assemblage (Romania: Haţeg Basin) - insight into a unique dinosaur nesting locality. Palaeogeogr. Palaeoclimatol. Palaeoecol. **468**, 228–262. (doi:10.1016/j.palaeo.2016.12.003)

Bravo AM, Gaete R. 2015 Titanosaur eggshells from the Tremp Formation (Upper Cretaceous, Southern Pyrenees, Spain). *Hist. Biol.* **27**, 1079–1089. (doi:10.1080/08912963.2014.934231)

Calvo JO, Engelland S, Heredia SE, Salgado L. 1997 First record of dinosaur eggshells (?Sauropoda - Megaloolithidae) from Neuquén, Patagonia, Argentina. *Gaia* **14**, 23–32.

Carpenter K, Alf K. 1994 Global distribution of dinosaur eggs, nests, and babies. In *Dinosaur Eggs and Babies* (eds K Carpenter, KF Hirsch, JR Horner), pp. 15–30. Cambridge, UK: Cambridge University Press.

Chassagne-Manoukian M, Haddoumi H, Cappetta H, Charriere A, Feist M, Tabuce R, Vianey-Liaud M. 2013 Dating the "red beds" of the Eastern Moroccan High Plateaus: evidence from late Late Cretaceous charophytes and dinosaur eggshells. *Geobios* **46**, 371–379. (doi:10.1016/j.geobios.2013.06.001)

Chiappe LM, Coria RA, Dingus L, Jackson F, Chinsamy A, Fox M. 1998 Sauropod dinosaur embryos from the Late Cretaceous of Patagonia. *Nature* **396**, 258–261.

Chiappe LM, Dingus L, Jackson FD, Grellet-Tinner G, Coria R, Loope D, Clarke J, Garrido A. 2001 Sauropod eggs and embryos from the Late Cretaceous of Patagonia. In *First International Symposium on Dinosaur Eggs and Babies Extended Abstracts* (eds AM Bravo, T Reyes), pp. 23–29. Catalonia, Spain.

Chimento NR, Agnolín FL, Manabe M, Tsuihiji T, Rich TH, Vickers-Rich P, Novas FE. 2023 First monotreme from the Late Cretaceous of South America. *Commun. Biol.* **6**, 146. (doi:10.1038/s42003-023-04498-7)

Chiappe LM, Schmitt JG, Jackson FD, Garrido A, Dingus L, Grellet-Tinner G. 2004 Nest structure for sauropods: sedimentary criteria for recognition of dinosaur nesting traces. *Palaios* **19**, 89–95. (doi:10.1669/0883-1351(2004)019<0089:NSFSSC>2.0.CO;2)

Codrea V, Smith T, Dica P, Folie A, Garcia G, Godefroit P, Van Itterbeeck J. 2002 Dinosaur egg nests, mammals and other vertebrates from a new Maastrichtian site of the Hateg Basin (Romania). *C. R. Palevol* **1**, 173–180. (doi:10.1016/S1631-0683(02)00021-0)

Company J. 2017 Unusually thick dinosaur eggshell fragments from the Spanish Late Cretaceous. *Hist. Biol.* **31**, 203–210. (doi:10.1080/08912963.2017.1357717)

Cousin R, Breton G, Fournier R, Watte J-P. 1989 Dinosaur egg-laying and nesting: the case of an Upper Maastrichtian site at Rennes-le-Chateau (Aude, France). *Hist. Biol.* **2**, 157–167. (doi:10.1080/08912968909386498)

Dhiman H, Dutta S, Kumar S, Verma V, Prasad GV. 2021 Discovery of proteinaceous moieties in Late Cretaceous dinosaur eggshell. *Palaeontology* **64**, 585–595. (doi:10.1111/pala.12565)

Dhiman H, Verma V, Prasad GV. 2022 First ovum-in-ovo pathological titanosaurid egg throws light on the reproductive biology of sauropod dinosaurs. *Sci. Rep.* **12**, 9362. (doi:10.1038/s41598-022-13257-3)

Dhiman H, Verma V, Singh LR, Miglani V, Jha DK, Sanyal P, Tandon SK, Prasad GV. 2023. New Late Cretaceous titanosaur sauropod dinosaur egg clutches from lower Narmada valley, India: palaeobiology and taphonomy. *PLoS ONE* **18**, e0278242. (doi:10.1371/journal.pone.0278242)

Diaz-Molina M, Kalin O, Benito MI, Lopez-Martinez N, Vicens E. 2007 Depositional setting and early diagenesis of the dinosaur eggshell-bearing Aren Fm at Bastus, Late Campanian, south-central Pyrenees. Sedimentary *Geology* **199**, 205–221. (doi:10.1016/j.sedgeo.2007.02.002)

Ensom PC. 1996 Reptile eggshell from the Purbeck Limestone Group of Dorset, Southern England. *Proc. Dorset Nat. Hist. Archaeol. Soc.* **118**, 79–83. (doi:10.1111/j.1365-2451.2007.00630.x)

Fang X, Lu L, Cheng Z, Zou Y, Pang Q, Wang Y, Chen K, Yin Z, Wang X, Liu J, Xie H, Jin Y. 1998 *On the Cretaceous Fossil Eggs of Xixia County, Henan Province*. Beijing, China: Geological Publishing House.

Fang X, Wang Y, Jiang Y. 2000 On the Late Cretaceous fossil eggs of Tiantai, Zhejiang. *Geol. Rev.* **46**, 107–112.

Fang X, Zhang Z, Zhang X, Lu L, Han Y, Li P. 2005 Fossil eggs from the Heyuan Basin, east-central Guangdong, China. *Geol. Bull. China* **24**, 682–686. (In Chinese with English abstract)

Fernández MS. 2014 Análisis de cáscaras de huevos de Dinosaurios de la Formación Allen, Cretácico Superior de Río Negro (Campaniano-Maastrichtiano): utilidad de los macrocaracteres de interés parataxonómico. *Ameghiniana* **50**, 79–97. (doi:10.5710/AMGH.14.11.2012.586) (In Spanish with English abstract)

Fernández MS, Khosla A. 2015 Parataxonomic review of the Upper Cretaceous dinosaur eggshells belonging to the oofamily Megaloolithidae from India and Argentina. *Hist. Biol.* **27**, 158–180. (doi:10.1080/08912963.2013.871718)

Fiorelli LE, Grellet-Tinner G, Alasino PH, Arganaraz E. 2012 The geology and palaeoecology of the newly discovered Cretaceous neosauropod hydrothermal nesting site in Sanagasta (Los Llanos Formation), La Rioja, northwest Argentina. *Cretaceous Res.* **35**, 94–117. (doi: 10.1016/j.cretres.2011.12.002)

Fiorelli LE, Martinelli AG, da Silva JI, Hechenleitner EM, Soares MVT, Silva Junior JC, da Silva JC, Borges EMR, Ribeiro LCB, Marconato A, Basilici G, da Silva Marinho T. 2022 First titanosaur dinosaur nesting site from the Late Cretaceous of Brazil. *Sci. Rep.* **12**, 5091. (doi:10.1038/s41598-022-09125-9)

Fondevilla V, Dinares-Turell J, Vila B, Le Loeuff J, Estrada R, Oms O, Galobart A. 2016 Magnetostratigraphy of the Maastrichtian continental record in the Upper Aude Valley (northern Pyrenees, France): Placing age constraints on the succession of dinosaur-bearing sites. *Cretaceous Res.* **57**, 457–472. (doi:10.1016/j.cretres.2015.08.009)

Garcia G. 2000 New data on the fossil eggshell diversity from the lower Maastrichtian of southern France. *Geobios* **33**, 113–126. (In French with English abstract)

Garcia G, Tabuce R, Cappetta H, Marandat B, Bentaleb I, Benabdallah A, Vianey-Liaud M. 2003 First record of dinosaur eggshells and teeth from the North-West African Maastrichtian (Morocco). *Palaeovertebrata* **32**, 59–69.

Garcia G, Vianey-Liaud M. 2001 Dinosaur eggshells as biochronological markers in Upper Cretaceous continental deposits. Palaeogeogr. Palaeoclimatol. Palaeoecol. **169**, 153–164. (doi:10.1016/S0031-0182(01)00215-2)

Genise JF, Sarzetti LC. 2011 Fossil cocoons associated with a dinosaur egg from Patagonia, Argentina. *Palaeontology* **54**, 815–823. (doi:10.1111/j.1475-4983.2011.01064.x)

Gottfried MD, O'Connor PM, Jackson RD, Roberts EM, Cham R. 2004 Dinosaur eggshell from the red sandstone group of Tanzania. *J. Vertebr. Paleont.* **24**, 494–497. (doi:10.1671/0272-4634(2004)024[0494:DEFTRS]2.0.CO;2)

Grellet-Tinner G, Chiappe LM, Coria R. 2004 Eggs of titanosaurid sauropods from the Upper Cretaceous of Auca Mahuevo (Argentina). *Can. J. Earth Sci.* **41**, 949–960. (doi:10.1139/E04-049)

Grellet-Tinner G, Fiorelli LE. 2010 A new Argentinean nesting site showing neosauropod dinosaur reproduction in a Cretaceous hydrothermal environment. *Nat. Commun.* **1**, 32. (doi:10.1038/Ncomms1031)

Grellet-Tinner G, Fiorelli LE, Salvador RB. 2012 Water vapor conductance of the Lower Cretaceous dinosaurian eggs from Sanagasta, La Rioja, Argentina: paleobiological and paleoecological implications for South American faveoloolithidand megaloolithid eggs. *Palaios* **27**, 35–47. (doi: 10.2110/palo.2011.p11-061r)

Grigorescu D. 2016 The ‘Tuştea puzzle’ revisited: Late Cretaceous (Maastrichtian) *Megaloolithus* eggs associated with *Telmatosaurus* hatchlings in the Haţeg Basin. *Hist. Biol.* **29**, 627–640. (doi:10.1080/08912963.2016.1227327)

Grigorescu D, Garcia G, Csiki Z, Codrea V, Bojar AV. 2010 Uppermost Cretaceous megaloolithid eggs from the Hateg Basin, Romania, associated with hadrosaur hatchlings: search for explanation. Palaeogeogr. Palaeoclimatol. Palaeoecol. **293**, 360–374. (doi:10.1016/j.palaeo.2010.03.031)

Grigorescu D, Weishampel DB, Norman D, Seclamen M, Rusu M, Baltres A, Teodorescu V. 1994 Late Maastrichtian dinosaur eggs from the Hateg Basin (Romania). In *Dinosaur Eggs and Babies* (eds K Carpenter, KF Hirsch, JR Horner), pp. 75–87. Cambridge, UK: Cambridge University Press.

Hechenleitner EM, Leuzinger L, Martinelli AG, Rocher S, Fiorelli LE, Taborda JR, Salgado L. 2020 Two Late Cretaceous sauropods reveal titanosaurian dispersal across South America. *Commun. Biol.* **3**, 622. (doi:10.1038/s42003-020-01338-w)

Huh M, Zelenitsky DK. 2002 Rich dinosaur nesting site from the Cretaceous of Bosung County, Chullanam-do Province, South Korea. *J. Vertebr. Paleontol.* **22**, 716–718. (doi:10.1671/0272-4634(2002)022[0716:RDNSFT]2.0.CO;2)

Jackson FD. 2007 *Titanosaur reproductive biology: comparison of the Auca Mahuevo titanosaur nesting locality (Argentina), to the Pinyes* Megaloolithus *nesting locality (Spain)*. Ph.D. Thesis. Bozeman: Montana State University.

Jackson FD, Schmitt JG, Oser SE. 2013 Influence of Vertisol development on sauropod egg taphonomy and distribution at the Auca Mahuevo locality, Patagonia, Argentina. Palaeogeogr. Palaeoclimatol. Palaeoecol. **386**, 300–307. (doi:10.1016/j.palaeo.2013.05.031)

Jackson FD, Varricchio DJ, Jackson RA, Vila B, Chiappe LM. 2008 Comparison of water vapor conductance in a titanosaur egg from the Upper Cretaceous of Argentina and a *Megaloolithus siruguei* egg from Spain. *Paleobiology* **34**, 229–246. (doi:10.1666/0094-8373(2008)034[0229:COWVCI]2.0.CO;2)

Jin X. 2013 *The Paleontological Study of Dinosaur and Turtle Fossil Eggs from Zhejiang Province, China*. Hanzhou, China: Zhejiang Science and Technology Publishing House.

Khosla A, Sahni A. 1995 Parataxonomic classification of Late Cretaceous dinosaur eggshells from India. *J. Palaeontol. Soc. India* **40**, 87–102.

Kim S, Hwang IG, Ghim YS, Kim NH, Lee YN. 2022 Upper Cretaceous (Coniacian-Santonian) dinosaur nesting colony preserved in abandoned crevasse splay deposits, Wi Island, South Korea. Palaeogeogr. Palaeoclimatol. Palaeoecol. **585**, 110728. (doi:10.1016/j.palaeo.2021.110728)

Kim SB, Kim YG, Jo HR, Jeong KS, Chough SK. 2009 Depositional facies, architecture and environments of the Sihwa Formation (Lower Cretaceous), mid-west Korea with special reference to dinosaur eggs. *Cretaceous Res.* **30**, 100–126. (doi:10.1016/j.cretres.2008.05.016)

Kim NH, Choi S, Kim S, Lee YN. 2019 A new faveoloolithid oogenus from the Wido Volcanics (Upper Cretaceous), South Korea and a new insight into the oofamily Faveoloolithidae. *Cretaceous Res.* **100**, 145–163. (doi:10.1016/j.cretres.2019.04.001)

Lee Y-N. 2003 Dinosaur bones and eggs in South Korea. *Mem. Fukui Prefect. Dinosaur Mus.* **2**, 113–121.

Lopez-Martinez N, Moratalla JJ, Sanz JL. 2000 Dinosaurs nesting on tidal flats. Palaeogeogr. Palaeoclimatol. Palaeoecol. **160**, 153–163. (doi:10.1016/S0031-0182(00)00063-8)

Lourembam RS, Dhiman H, Prasad GV. 2023 Preservation of Membrana Testacea in titanosaurid dinosaur eggshells from the Upper Cretaceous Deccan volcano-sedimentary strata of Central India. *Geol. Mag.* **160**, 361–371. (doi:10.1017/S0016756822000978)

Mikhailov K. 1994 Eggs of sauropod and ornithopod dinosaurs from the Cretaceous deposits of Mongolia. *Paleontol. J.* **28**, 141–159.

Mohabey DM. 1984 The study of dinosaurian eggs from infratrappean limestone in Kheda District, Gujarat. *J. Geol. Soc. India* **25**, 329–337. (doi:10.17491/jgsi/1984/250601)

Mohabey DM. 1996 A new oospecies, *Megaloolithus matleyi*, from the Lameta Formation (Upper Cretaceous) of Chandrapur district, Maharashtra, India, and general remarks on the palaeoenvironment and nesting behaviour of dinosaurs. *Cretaceous Res.* **17**, 183–196. (doi:10.1006/cres.1996.0014)

Mohabey DM. 1998 Systematics of Indian Upper Cretaceous dinosaur and chelonian eggshells. *J. Vertebr. Paleontol.* **18**, 348–362. (doi:10.1080/02724634.1998.10011063)

Mohabey DM, Mathur UB, 1989 Upper Cretaceous dinosaur eggs from new localities of Gujarat, India. *J. Geol. Soc. India* **33**, 32–37. (doi:10.17491/jgsi/1989/330103)

Mones A. 1980 Nuevos elementos de la paleoherpetofauna del uruguay (Crocodilia y Dinosauria). *Actas II Congr. Argent. Paleontol. Bioestratigr. I Congr. Latinoam. Paleontol.* **2-6 de abril, 1978, 1**, 265–278. Buenos Aires, Argentina. (In Spanish with English abstract)

Paik IS, Huh M, Kim HJ. 2004 Dinosaur egg-bearing deposits (Upper Cretaceous) of Boseong, Korea: occurrence, palaeoenvironments, taphonomy, and preservation. Palaeogeogr. Palaeoclimatol. Palaeoecol. **205**, 155–168. (doi:10.1016/j.palaeo.2003.12.007)

Paik IS, Kim HJ, Huh M. 2012 Dinosaur egg deposits in the Cretaceous Gyeongsang Supergroup, Korea: diversity and paleobiological implications. *J. Asian Earth Sci.* **56**, 135–146. (doi:10.1016/j.jseaes.2012.05.006)

Peitz C. 2001 Megaloolithid dinosaur eggs from the Maastrichtian of Catalunya (NE-Spain) -parataxonomic implications and stratigraphic utility. In *First International Symposium on Dinosaur Eggs and Babies Extended Abstracts* (eds AM Bravo, T Reyes), pp. 155–160. Catalonia, Spain.

Powell, JE. 1987 The Late Cretaceous fauna of Los Alamitos, Patagonia, Argentina. VI. Los Titanosauridae. *Rev. Mus. Argent. Cienc. Nat. Bernardino Rivadavia* **3**, 148-153.

Ribeiro CMM. 2002 Ovo e fragmentos de cascas de ovos de dinossauros, provenientes da regiao de Peiropolis, Uberaba, Minas Gerais. *Arq. Mus. Nac.* **60**, 223–228. (In Portuguese with English abstract)

Salgado L, Coria RA, Ribeiro CMM, Garrido A, Rogers R, Simon ME, Arcucci AB, Rogers KC, Carabajal AP, Apesteguia S, Fernández M, Garcia RA, Talevi M. 2007 Upper Cretaceous dinosaur nesting sites of Rio Negro (Salitral Ojo de Agua and Salinas de Trapalco-Salitral de Santa Rosa), northern Patagonia, Argentina. *Cretaceous Res.* **28**, 392–404. (doi:10.1016/j.cretres.2006.06.007)

Salgado L, Ribeiro CM, Garcia RA, Fernández MS. 2009 Late Cretaceous megaloolithid eggs from Salitral de SantaRosa (Rio Negro, Patagonia, Argentina): inferences on the titanosaurian reproductive biology. *Ameghiniana* **46**, 605–620.

Sander PM, Peitz C, Gallemi J, Cousin R. 1998 Dinosaurs nesting on a red beach? *C. R. Acad. Sci. Ser. IIa-Earth Planet. Sci.* **327**, 67–74. (doi:10.1016/S1251-8050(98)80020-7)

Sanguino F, Celis AD, Horra Del Barco RDL, Fernández E, Fernández Martínez J, Marcos Fernández MF, Adan Perez-García, Ortega F. 2025 A unique association of fusioolithid dinosaur eggs from the Upper Cretaceous of Spain (Poyos, central system). *Cretaceous Res.* **174**, 106122. (doi:10.1016/j.cretres.2025.106122)

Sanz JL, Moratalla JJ, Diazmolina M, Lopezmartinez N, Kalin O, Vianeyliaud M. 1995 Dinosaur nests at the sea shore. *Nature* **376**, 731–732. (doi:10.1038/376731a0)

Sellés AG, Bravo AM, Delclos X, Colombo F, Marti X, Ortega-Blanco J, Parellada C, Galobart A. 2013 Dinosaur eggs in the Upper Cretaceous of the Coll de Nargo area, Lleida Province, south-central Pyrenees, Spain: oodiversity, biostratigraphy and their implications. *Cretaceous Res.* **40**, 10–20. (doi:10.1016/j.cretres.2012.05.004)

Simon ME. 2006 Cáscaras de huevos de dinosaurios de la Formación Allen (Campaniano-Maastrichtiano), en Salitral Moreno, provincia de Río Negro, Argentina. *Ameghiniana* **43**, 513–528. (In Spanish with English abstract)

Smith T, Codrea VA, Sasaran E, Van Itterbeeck J, Bultynck P, Csiki Z, Dica P, Farcas C, Folie A, Garcia G, Godefroit P. 2002 A new exceptional vertebrate site from the Late Cretaceous of the Hateg Basin (Romania). *Studia Univ. Babes-Bolyai, Geol., Special Issue* **1**, 321–330.

Sochava AV. 1969 Dinosaur eggs from the Upper Cretaceous of the Gobi Desert. *Paleontol. J.* **4**, 517–527.

Soto M, Perea D, Cambiaso A. 2012 First sauropod (Dinosauria: Saurischia) remains from the Guichon Formation, Late Cretaceous of Uruguay. *J. S. Am. Earth Sci.* **33**, 68–79. (doi:10.1016/j.jsames.2011.08.003)

Srivastava AK, Mankar RS. 2015 *Megaloolithus* dinosaur nest from the Lameta Formation of Salbardi area, districts Amravati, Maharashtra and Betul, Madhya Pradesh. *J. Geol. Soc. India* **85**, 457–462. (doi:10.1007/s12594-015-0237-0)

Srivastava S, Mohabey DM, Sahni A, Pant SC. 1986 Upper Cretaceous dinosaur egg clutches from Kheda District (Gujarat, India). *Palaeontogr. Abt. A* **193**, 219–233.

Venczel M, Vasile S, Csiki-Sava Z. 2015 A Late Cretaceous madtsoiid snake from Romania associated with a megaloolithid egg nest -paleoecological inferences. *Cretaceous Res.* **55**, 152–163. (doi:10.1016/j.cretres.2015.02.009)

Vianey-Liaud M, Hirsch KE, Sahni A, Sige B. 1997 Late Cretaceous Peruvian eggshells and their relationships with Laurasian and Eastern Gondowanian material. *Geobios* **30**, 75–90. (doi:10.1016/S0016-6995(97)80259-8)

Vianey-Liaud M, Khosla A, Garcia G. 2003 Relationships between European and Indian dinosaur eggs and eggshells of the oofamily Megaloolithidae. *J. Vertebr. Paleontol.* **23**, 575–585. (doi:10.1671/0272-4634(2003)023[0575:RBEAID]2.0.CO;2)

Vianey-Liaud M, Lopez-Martinez N. 1997 Late Cretaceous dinosaur eggshells from the Tremp Basin, southern Pyrenees, Lleida, Spain. *J. Paleontol.* **71**, 1157–1171. (doi:10.1017/S002233600003609X)

Vianey-Liaud M, Mallan P, Buscail O, Montgelard C. 1994 Review of French dinosaur eggshells: morphology, structure, mineral, and organic composition. In *Dinosaur Eggs and Babies* (eds K Carpenter, KF Hirsch, JR Horner), pp. 151–183. Cambridge, UK: Cambridge University Press.

Vila B, Galobart À, Oms O, Poza B, Bravo AM. 2010b Assessing the nesting strategies of Late Cretaceous titanosaurs: 3-D clutch geometry from a new megaloolithid eggsite. *Lethaia* **43**, 197–208. (doi:10.1111/j.1502-3931.2009.00183.x)

Vila B, Jackson FD, Fortuny J, Sellés AG, Galobart À. 2010a 3-D modelling of megaloolithid clutches: insights about nest construction and dinosaur behaviour. *PLoS ONE* **5**, e10362. (doi:10.1371/journal.pone.0010362)

Wang Q, Zhao Z, Wang XL, Jiang Y. 2011 New ootypes of dinosaur eggs from the Late Cretaceous in Tiantai Basin, Zhejiang Province, China. *Vertebr. PalAsiatica* **49**, 446–449.

Wang X-L, Wang Q, Jiang S-X, Cheng X, Zhang J-L, Zhao Z-K, Jiang Y-G. 2012 Dinosaur egg faunas of the Upper Cretaceous terrestrial red beds of China and their stratigraphical significance. *J. Stratigr.* **36**, 400–416. (doi:10.19839/j.cnki.dcxzz.2012.02.017)

Watabe M, Tsogtbaatar K, Suzuki S, Saneyoshi M. 2010 Geology of dinosaur-fossil-bearing localities (Jurassic and Cretaceous: Mesozoic) in the Gobi Desert: results of the HMNS-MPC Joint Paleontological Expedition. *Hayashibara Mus. Nat. Sci. Res. Bull.* **3**, 41–118.

Williams DLG, Seymour RS, Kerourio P. 1984 Structure of fossil dinosaur eggshell from the Aix Basin, France. Palaeogeogr. Palaeoclimatol. Palaeoecol. **45**, 23–37. (doi:10.1016/0031-0182(84)90107-X)

Wilson JA, Mohabey DM, Peters SE, Head JJ. 2010 Predation upon hatchling dinosaurs by a new snake from the Late Cretaceous of India. *PLoS Biol.* **8**, e1000322. (doi:10.1371/journal.pbio.1000322)

Zhang S-K. 2010 A parataxonomic revision of the Cretaceous faveoloolithid eggs of China. *Vertebr. PalAsiatica* **48**, 203–219.

Zhao Z, Ding S. 1976 Discovery of the dinosaurian eggshells from Alxa, Ningxia and its stratigraphic significance. *Vertebr. PalAsiatica* **14**, 42–44. (In Chinese)

Zheng T, Bai Y, Wang Q, Zhu X, Fang K, Yao Y, Zhao Y, Wang X. 2018 A new ootype of dinosaur egg (Faveoloolithidae: *Duovallumoolithus shangdanensis* oogen. et oosp. nov.) from the Late Cretaceous in the Shangdan Basin, Shaanxi Province, China. *Acta Geol. Sin.* **92**, 897–903. (doi:10.1111/1755-6724.13581)

Zhou S, Cui B, Guo Y, Qian X, Jin H. 2010 *The World of Dinosaur Eggs in Zoujin*. Wuhan, China: China University of Geoscience Press. (In Chinese)

Zhou X, Ren Y, Xu S, Guan K. 1998 Dinosaur eggs of the Late Cretaceous from the Qinglongshan region, Yunxian County, Hubei Province. *Hubei Geol. Miner. Resour.* **12**, 1–8. (In Chinese with English abstract)

Zou SL, Wang Q, Wang XL. 2013 A new oospecies of parafaveoloolithids from the Pingxiang Basin, Jiangxi Province of China. *Vertebr. PalAsiatica* **51**, 102–106.
